# Tracing the evolution of single-cell 3D genomes in Kras-driven cancers

**DOI:** 10.1038/s41588-025-02297-w

**Published:** 2025-08-18

**Authors:** Miao Liu, Shengyan Jin, Sherry S. Agabiti, Tyler B. Jensen, Tianqi Yang, Jonathan S. D. Radda, Christian F. Ruiz, Gabriel Baldissera, Moein Rajaei, Fang-Yong Li, Jeffrey P. Townsend, Mandar Deepak Muzumdar, Siyuan Wang

**Affiliations:** 1 Department of Genetics, Yale School of Medicine, Yale University, New Haven, CT USA; 2 Yale Cancer Biology Institute, Yale University, West Haven, CT USA; 3 M.D.-Ph.D. Program, Yale University, New Haven, CT USA; 4 Department of Biostatistics, Yale School of Public Health, Yale University, New Haven, CT USA; 5 Department of Chronic Disease Epidemiology, Yale School of Public Health, Yale University, New Haven, CT USA; 6 Yale Cancer Center, Yale University, New Haven, CT USA; 7 Program in Computational Biology and Bioinformatics, Yale University, New Haven, CT USA; 8 Program in Genetics, Genomics, and Epigenetics, Yale Cancer Center, Yale University, New Haven, CT USA; 9 Department of Internal Medicine, Section of Medical Oncology, Yale School of Medicine, Yale University, New Haven, CT USA; 10 Yale Combined Program in the Biological and Biomedical Sciences, Yale University, New Haven, CT USA; 11 Molecular Cell Biology, Genetics, and Development Program, Yale University, New Haven, CT USA; 12 Department of Cell Biology, Yale School of Medicine, Yale University, New Haven, CT USA; 13 Biochemistry, Quantitative Biology, Biophysics, and Structural Biology Program, Yale University, New Haven, CT USA; 14 Yale Center for RNA Science and Medicine, Yale School of Medicine, Yale University, New Haven, CT USA; 15 Yale Liver Center, Yale School of Medicine, Yale University, New Haven, CT USA

**Keywords:** Epigenomics, Non-small-cell lung cancer, Molecular imaging, Tumour biomarkers, Gene regulation

## Abstract

Although three-dimensional (3D) genome structures are altered in cancer, it remains unclear how these changes evolve and diversify during cancer progression. Leveraging genome-wide chromatin tracing to visualize 3D genome folding directly in tissues, we generated 3D genome cancer atlases of oncogenic Kras-driven mouse lung adenocarcinoma (LUAD) and pancreatic ductal adenocarcinoma. Here we define nonmonotonic, stage-specific alterations in 3D genome compaction, heterogeneity and compartmentalization as cancers progress from normal to preinvasive and ultimately to invasive tumors, discovering a potential structural bottleneck in early tumor progression. Remarkably, 3D genome architectures distinguish morphologic cancer states in single cells, despite considerable cell-to-cell heterogeneity. Analyses of genome compartmentalization changes not only showed that compartment-associated genes are more homogeneously regulated but also elucidated prognostic and dependency genes in LUAD, as well as an unexpected role for Rnf2 in 3D genome regulation. Our results highlight the power of single-cell 3D genome mapping to identify diagnostic, prognostic and therapeutic biomarkers in cancer.

## Abstract (web summary)

This study uses chromatin tracing to identify alterations in single-cell 3D genome conformation during the progression of Kras-driven mouse lung adenocarcinoma and pancreatic ductal adenocarcinoma, and proposes Rnf2 as a regulator of the 3D genome.

## Main

Cancer cells exhibit profound alterations in nuclear size, shape and chromatin texture^1^—features that are conventionally used for diagnosis and establishing pathologic cancer grade^1^. It has become increasingly clear that the three-dimensional (3D) genome organization within the nucleus has a crucial role in both normal physiology and disease^2–9^. Here we present genome-wide, in situ single-cell cancer 3D genome atlases, generated using an imaging-based 3D genomics method that we have pioneered—chromatin tracing^10,11^. Unlike sequencing-based 3D genomics technologies (for example, high-throughput chromosome conformation capture (Hi-C)) that relied on population averaging of cells and indirect inference of altered genome organization in averaged cancer cells^12–17^, our approach captures 3D genome folding directly in individual cells in the native tissue environment in vivo during cancer progression from normal to preinvasive and to invasive tumor cells. Our results also define stage-specific alterations in 3D genome architectures during lung adenocarcinoma (LUAD) and pancreatic ductal adenocarcinoma (PDAC) progression and illustrate how 3D genome evolution can nominate genes that govern prognosis, delineate cancer cell dependency and regulate the 3D genome. Our findings and methodologies offer a unique resource by which in situ single-cell 3D genome information helps to identify potential diagnostic, prognostic and therapeutic cancer biomarkers.

## Results

### Genome-wide chromatin tracing in mouse LUAD

**Fig. 1:**
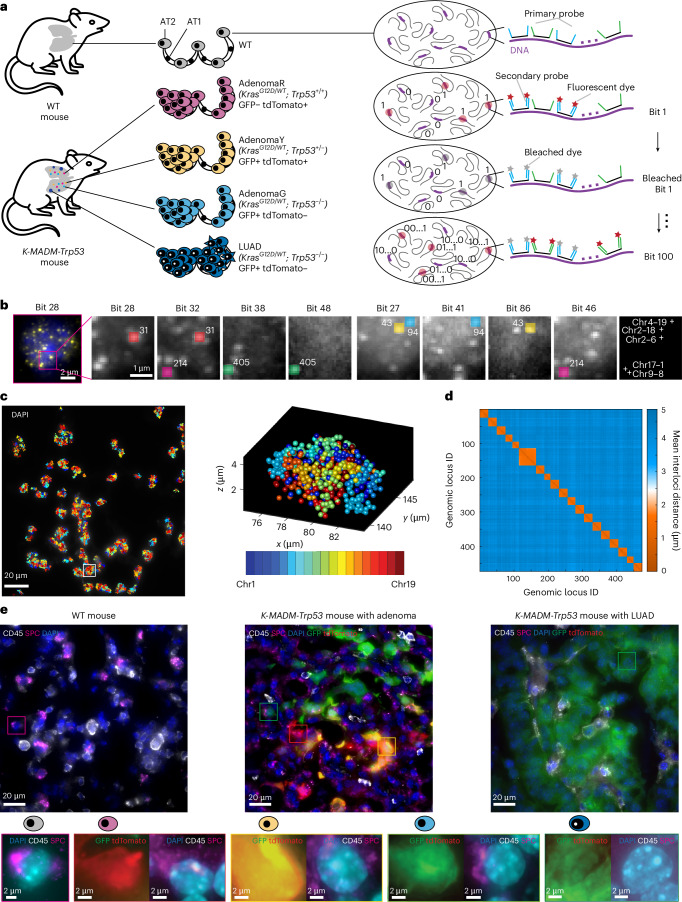
A genome-scale chromatin tracing strategy to visualize cancer 3D genomes. **a**, Schematic illustration of the experimental procedure. **b**, Raw FISH foci (left) in bit 28 in a WT AT2 cell. Zoom-in images (right) of raw FISH foci showing the decoding procedure and reconstructed genomic loci for five loci (shown as dots with five pseudocolors each appearing twice). Representative results from five biological replicates (*n* = 4 mice) are shown in **b** and **c**. **c**, Reconstructed chromatin traces (left) superimposed with DAPI staining. The traces are 2D projections of *x* and *y* coordinates. The DAPI image is a maximum-intensity *z* projection from a 10-μm z stack. The 3D positions (right) of all decoded genomic loci in a single-cell nucleus. Different pseudocolors represent different autosomes. **d**, Matrix of mean interloci distances between all genomic loci in AT2 cells. *n* = 4,806 AT2 cells. **e**, Immunofluorescence staining (top) of cell-type markers, DAPI staining and fluorescent protein imaging of lung tissue from a WT mouse, a *K-MADM-Trp53* mouse with adenomas and a *K-MADM-Trp53* mouse with LUAD. Representative results from five biological replicates in WT (*n* = 4 mice) and adenoma (*n* = 4 mice) and six biological replicates in LUAD (*n* = 5 mice) states are shown. Representative cells (bottom) of each state. The images are maximum-intensity *z* projections from 10-μm z stacks. Chr, chromosome. Source data

**Extended Data Fig. 1:**
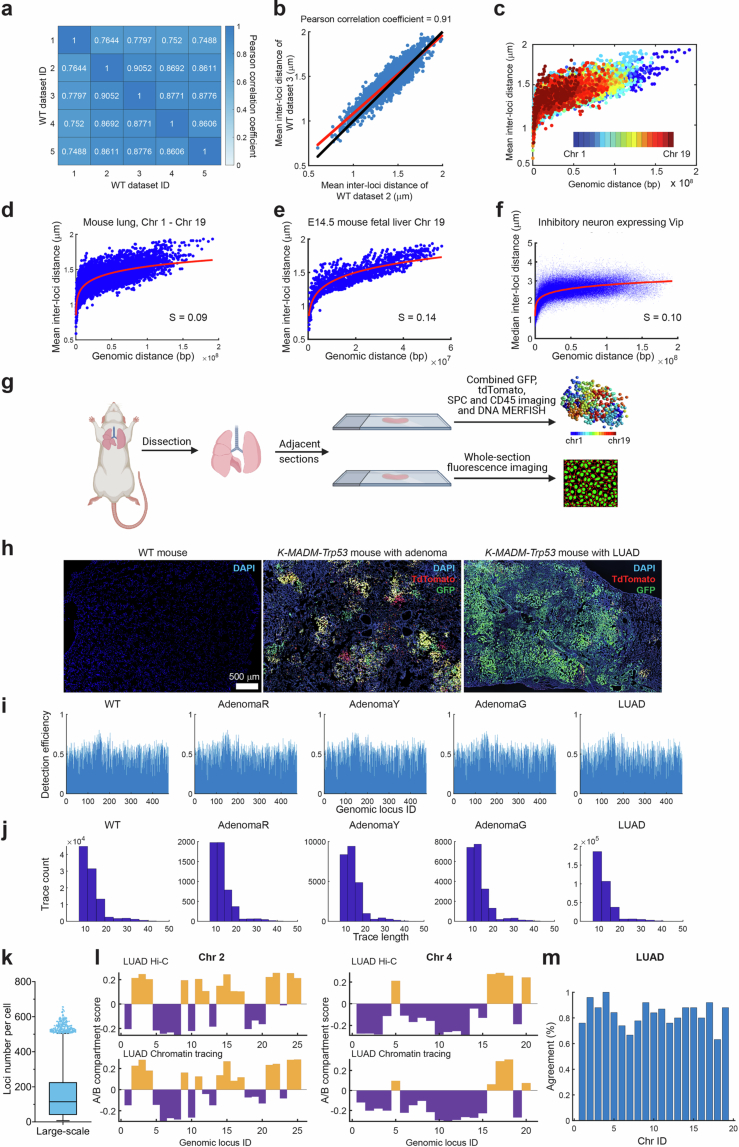
Genome-scale chromatin tracing visualizes 3D genome organization in vivo. **a**, Pearson correlation coefficients of mean *cis*-chromosomal interloci distances between WT datasets. **b**, Mean *cis*-chromosomal interloci distances of WT dataset 3 versus mean *cis*-chromosomal interloci distances of WT dataset 2. The red line is a fitted linear regression line. The black line is the *y* = *x* line. **c**, Mean *cis*-chromosomal interloci spatial distance versus genomic distance for all pairs of genomic loci on each autosome in AT2 cells. Different pseudocolors represent different autosomes. *n* = 6,039 intrachromosomal interloci pairs in **b**–**d**. *n* = 4,806 WT AT2 cells in **c** and **d**. **d**, Power-law scaling of all 19 mouse autosomes (chr1–19) in WT mouse lung. **e**, Power-law scaling of chr19 in E14.5 mouse fetal liver. Data were re-analyzed from ref. ^24^. **f**, Power-law scaling of all 20 mouse chromosomes (chr1–19, chrX) in the mouse brain inhibitory neurons expressing Vip. Data were re-analyzed from ref. ^23^. **g**, Schematic illustration of the experimental procedure. The schematic in panel **g** was created with BioRender.com. **h**, Whole-section fluorescence images of WT mouse lung and *K-MADM-Trp53* mouse lungs containing adenomas or LUAD. Results are representative of 5 biological replicates in WT (4 mice) and adenoma (4 mice) states, and 6 biological replicates in the LUAD (5 mice) state. **i**, Detection efficiency of each target genomic region along detected chromosome traces in each cancer state. **j**, Distribution of the trace length across individual chromosomes in cells of each cancer state. **k**, Distribution of detected loci number per cell. *n* = 26,852 cells combining the five cell states. The horizontal lines of the box from top to bottom represent the 75th percentile, the median and the 25th percentile. Whiskers extend to the nonoutlier maximum and nonoutlier minimum. Outliers are defined as values at least 1.5 times interquartile range away from the top or bottom of the box. **l**, A and B compartment score profiles of example autosomes measured by Hi-C and chromatin tracing in mouse LUAD. **m**, Fraction of genomic regions with concordant A and B compartment assignment between Hi-C and chromatin tracing. For **d**–**f**, lines are fitted power-law functions, and S is the scaling factor. Source data

To directly visualize chromatin folding in single cells within tissues, we performed genome-wide chromatin tracing targeting 473 genomic loci spanning all 19 mouse autosomes at an average genomic interval of 5 megabases (Mb) (Supplementary Tables 1–3). These genomic regions contained ‘classic’ oncogenes, tumor suppressors and super-enhancers. We adapted a DNA multiplexed error-robust fluorescence in situ hybridization (DNA MERFISH) 100-choose-2 combinatorial barcoding design^18^ as follows: each locus was assigned a unique 100-bit binary barcode with two ‘1’ bits and 98 ‘0’ bits^18^; each ‘1’ bit physically corresponded to a unique overhang readout sequence on primary probes hybridized to the genome (Fig. 1a). The primary probes targeting each genomic locus contained two versions of readout sequences that corresponded to the two ‘1’ bits in the barcode of the locus. After hybridizing all the primary probes, we sequentially hybridized dye-labeled readout probes to image the 100 bits, with 50 rounds of two-color imaging (Fig. 1a). We then fitted the 3D positions of the foci imaged by fluorescence in situ hybridization (FISH), and decoded the barcodes and reconstructed chromatin traces in single cells (Fig. 1b,c). To validate the quality of genome-wide chromatin tracing, we also measured the mean spatial distance among each pair of target genomic loci and constructed a mean interloci distance matrix (Fig. 1d). We first performed this in lung alveolar type 2 (AT2) cells—the putative cell of origin for LUAD^19–21^—in wild-type (WT) mice. The resulting matrix featured lower intrachromosomal distances and higher interchromosomal distances, consistent with chromosome territory organization^22^. Notably, intrachromosomal interloci distances measured from WT AT2 biological replicates were highly reproducible (Extended Data Fig. 1a,b). Within each chromosome, interloci spatial distances scaled with expected genomic distances, with longer chromosomes reaching larger spatial distances (Extended Data Fig. 1c). On average, intrachromosomal interloci spatial distances followed a power-law function versus genomic distances, with a scaling factor of about one-tenth (Extended Data Fig. 1d), in line with prior studies (Extended Data Fig. 1e,f)^23,24^.

Having established the approach in normal tissues, we next performed chromatin tracing on the *K-MADM-Trp53* mouse lung cancer model (Fig. 1a and Extended Data Fig. 1g,h) that induces sparse and sequential *Kras* and *Trp53* mutations in the lung epithelium to mimic the faithful genetic and histologic progression of human LUAD^25–27^. Specifically, we leveraged the mosaic analysis with double markers (MADM) system^28^ to unambiguously link genotypes with fluorescence labeling^28,29^. Subclones of green (green fluorescent protein, GFP+) *Trp53*^−/−^, red (tdTomato+) *Trp53*^*+/+*^ and yellow (GFP+/tdTomato+) *Trp53*^*+/−*^ cells can be traced in preinvasive *Kras* mutant lung adenomas initiated by inhaled lentiviral Cre infection^30^. Only *Trp53*^−/−^, and not *Trp53*^*+/+*^ or *Trp53*^*+/−*^ adenoma cells, progressed to advanced LUAD^30^, enabling in vivo tracking of morphologically distinct stages from AT2 to preinvasive adenoma to LUAD. We combined multiplexed fluorescence imaging of GFP, tdTomato, surfactant protein C (SPC) and CD45 with genome-wide chromatin tracing on the same tissue section to achieve cancer state-specific single-cell 3D genome profiling (Extended Data Fig. 1g,h). SPC marks AT2 and cancer cells, whereas the pan-immune marker CD45 allows us to exclude macrophages, which are labeled alongside cancer cells by the MADM system (presumably through lentiviral Cre infection; Fig. 1e).

To build a complete 3D genome atlas capturing the full spectrum of tumor development, we analyzed 26,852 cells from 16 replicates across multiple lung lobes (most containing several discrete tumors) of 13 mice harboring all stages of LUAD progression which are as follows: AT2, AdenomaR (red *Trp53*^*+/+*^), AdenomaY (yellow *Trp53*^*+/−*^), AdenomaG (green *Trp53*^−/−^) and LUAD cells. The detection efficiency of each target region and chromosome trace length distribution were consistent across cell states (Extended Data Fig. 1i,j). Overall, we detected a median of 115 loci per cell (Extended Data Fig. 1k). A (active) and B (inactive) compartment assignments from chromatin tracing correlated with those from bulk Hi-C of LUAD tumors (Extended Data Fig. 1l,m). Together, these metrics validated the utility of chromatin tracing to compare chromatin conformations across cell states during LUAD progression.

### A 3D genome bottleneck in lung adenoma

**Fig. 2:**
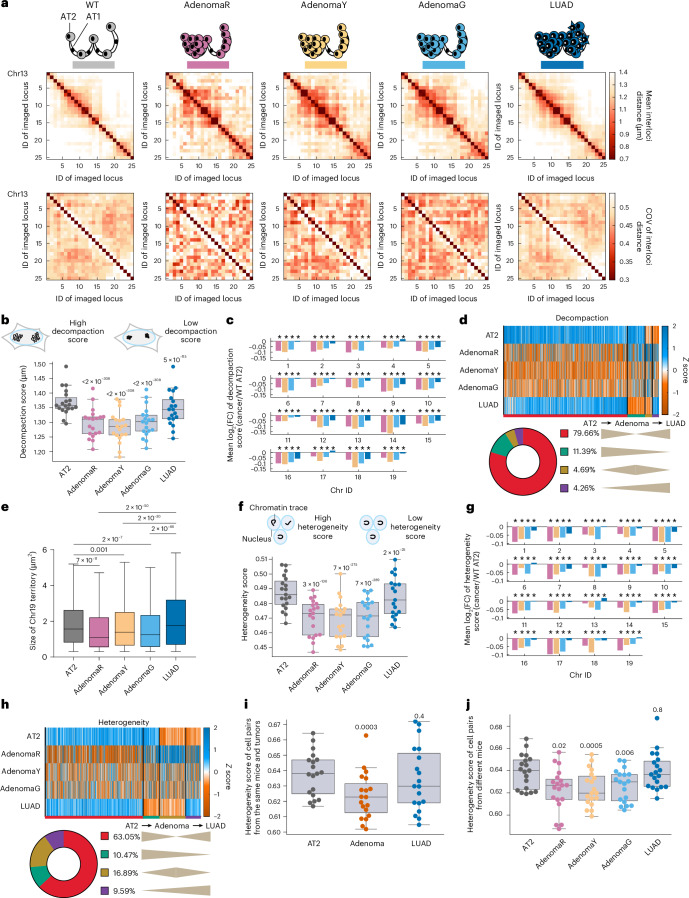
Systematic changes of 3D genome compaction and heterogeneity during lung cancer progression. **a**, Mean interloci distances (top) and COV of interloci distances (bottom) of mouse chr13 in WT AT2 (*n* = 4,806), *Trp53*^*+/+*^ adenoma (AdenomaR, *n* = 791), *Trp53*^*+/−*^ adenoma (AdenomaY, *n* = 1,603), *Trp53*^*−/−*^ adenoma (AdenomaG, *n* = 1,941) and *Trp53*^*−/−*^ LUAD (*n* = 17,711) cells. Cell numbers are identical in **a**–**j**. **b**, Distributions of decompaction (mean interloci distance) scores of each autosome (*n* = 19 autosomes) in each cell state. *P* values from two-sided Wilcoxon signed-rank tests versus AT2 with matched genomic loci are displayed. **c**, Mean log_2_(FC) of decompaction scores of each chromosome, comparing each cancer state to AT2. An asterisk indicates FDR < 0.05, two-sided Wilcoxon signed-rank test. **d**, Clustered heatmap of *z* scores (scaled by column) of *cis*-chromosomal mean interloci distances. Percentages of locus pairs with different decompaction change patterns are shown below. **e**, Chr19 territory size measured by whole chromosome paint. *P* values from one-sided Wilcoxon rank-sum tests are shown. **f**, Distributions of heterogeneity (COV of interloci distance) scores of each autosome (*n* = 19) in each cell state. *P* values from two-sided Wilcoxon signed-rank tests versus AT2 with matched genomic loci are displayed. **g**, Mean log_2_(FC) of heterogeneity scores of each chromosome, comparing each cancer state to AT2. An asterisk indicates FDR < 0.05, two-sided Wilcoxon signed-rank test. **h**, Clustered heatmap of *z* scores (scaled by column) of COV of *cis*-chromosomal interloci distances. Percentages of locus pairs with different chromatin folding heterogeneity change patterns are shown below. **i**,**j**, Distributions of 3D genome heterogeneity scores of autosomes (*n* = 19 autosomes) for cell pairs from the same mice (AT2, *n* = 4 mice) or same tumors (12 adenoma tumors and 6 LUAD tumors, intramouse or intratumoral; **i**) or from different mice (intermouse or intertumoral; **j**). *P* values from two-sided Wilcoxon signed-rank tests versus AT2 with matched autosomes are shown. In **b**, **e**, **f**, **i** and **j**, horizontal lines of each box represent the 75th percentile, median and 25th percentile. Whiskers indicate nonoutlier maximum and nonoutlier minimum, with outlier thresholds at 1.5× interquartile range beyond box limits. FC, fold change. Source data

**Extended Data Fig. 2:**
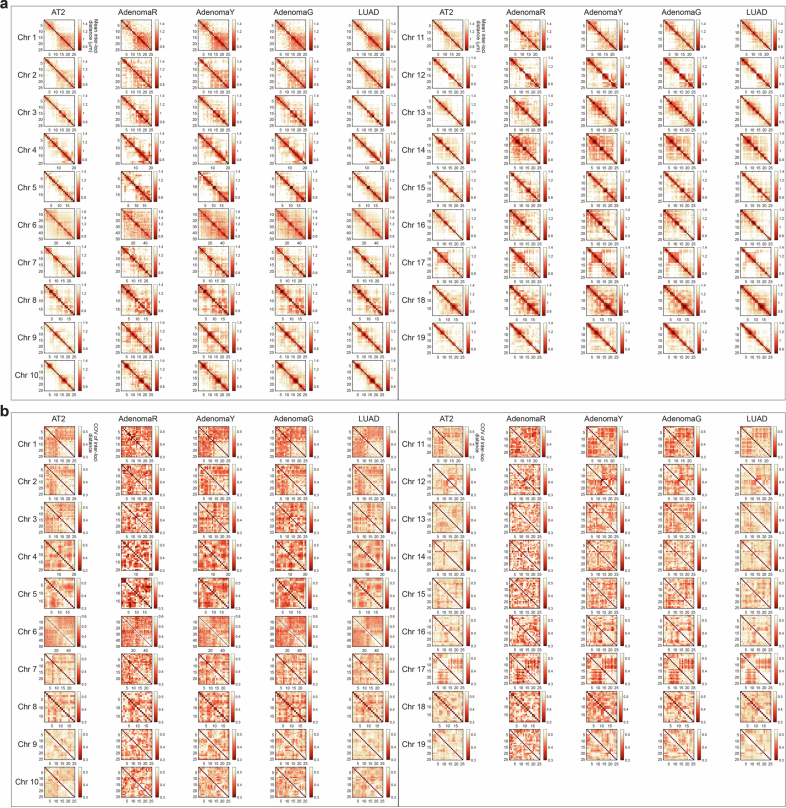
Systematic changes of intrachromosomal compaction and chromatin conformation heterogeneity during lung cancer progression. **a**,**b**, Mean interloci distances (**a**) and coefficients of variation (COV) of interloci distances (**b**) of all autosomes in WT AT2 (*n* = 4,806), *Trp53*^*+/+*^ adenoma (AdenomaR, *n* = 791), *Trp53*^*+/−*^ adenoma (AdenomaY, *n* = 1,603), *Trp53*^*−/−*^ adenoma (AdenomaG, *n* = 1,941) and *Trp53*^*−/−*^ LUAD (*n* = 17,711) cells. Source data

**Extended Data Fig. 3:**
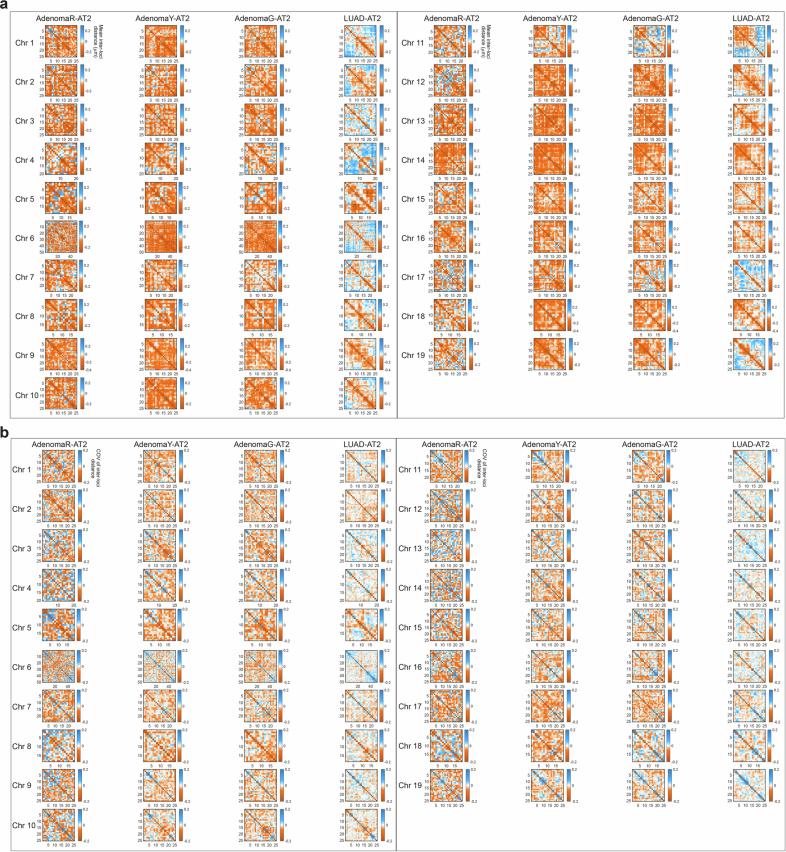
Subtraction maps showing systematic differences of intrachromosomal compaction and chromatin conformation heterogeneity during lung cancer progression. **a**,**b**, Subtraction maps of mean interloci distances (**a**) and coefficients of variation (COV) of interloci distances (**b**) in all autosomes of *Trp53*^*+/+*^ adenoma (AdenomaR, *n* = 791), *Trp53*^*+/−*^ adenoma (AdenomaY, *n* = 1,603), *Trp53*^*−/−*^ adenoma (AdenomaG, *n* = 1,941) and *Trp53*^*−/−*^ LUAD (*n* = 17,711) cells minus those of WT AT2 (*n* = 4,806) cells. Source data

To study how the 3D genome evolves during LUAD progression, we first quantified intrachromosomal interloci distances to assess chromatin compaction in each cell state (AT2, AdenomaR, AdenomaY, AdenomaG and LUAD). Intrachromosomal compaction globally increased during early progression from AT2 to preinvasive adenoma cells at the population level and recovered with the transition to LUAD (Fig. 2a–c and Extended Data Fig. 2a). Approximately 80% of locus pairs followed this nonmonotonic trend (Fig. 2d). As an orthogonal approach, chromosome paint of mouse chromosome 19 showed consistently greater whole chromosome compaction in adenoma cells compared to AT2 and LUAD cells (Fig. 2e). Strikingly, single-cell comparisons of chromatin traces revealed that chromatin conformations in adenoma cells were globally less heterogeneous than WT AT2 and LUAD cells (Fig. 2a,f,g and Extended Data Fig. 2b) with more than 60% locus pairs exhibiting this trend (Fig. 2h). Notably, diminished heterogeneity in the adenoma state was observed both within individual mice (for AT2 cells) or tumors (for adenoma and LUAD) and across mice (Fig. 2i,j), arguing for phenotypic convergence of chromatin compaction during early lung cancer progression. Furthermore, the recovery of heterogeneity and especially compaction in LUAD cells diverged from the AT2 state for many locus pairs (Extended Data Fig. 3), suggesting that locus-specific 3D genomic changes may confer a selective advantage in LUAD progression.

**Fig. 3:**
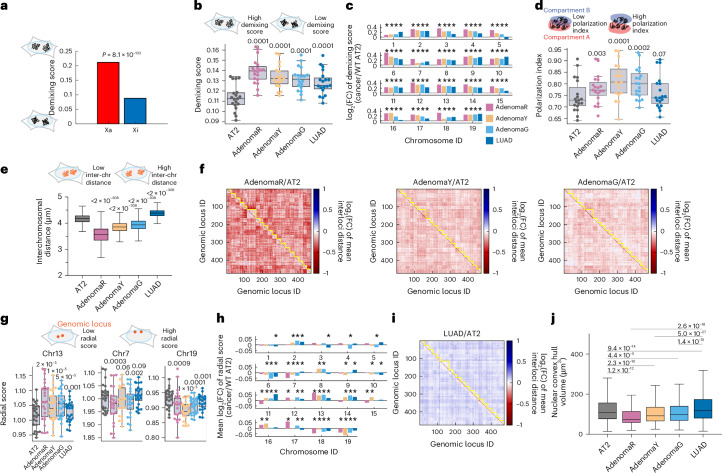
Changes of cancer state-specific 3D genome features during lung cancer progression. **a**, Demixing scores (s.d. of normalized mean interloci distances) of the active chrX (X_*a*_, *n* = 95) and inactive chrX (X_*i*_, *n* = 95) in human IMR90 cells show a reduction (increased intermixing) in X_*i*_, as previously described using other analyses^11^. *P* value from two-sided Levene’s test is shown. **b**, Distributions of demixing scores of each autosome (*n* = 19) in each cell state. *n* = 4,806, 791, 1,603, 1,941 and 17,711 for WT AT2, AdenomaR, AdenomaY, AdenomaG and LUAD cells, respectively. Cell numbers of each cell state are identical in **b**–**i**. **c**, log_2_(FC) of the demixing score of each autosome, comparing each cancer state to WT AT2 cell state. An asterisk indicates FDR < 0.05, two-sided Levene’s test. **d**, Distribution of polarization indices of each autosome (*n* = 19) in each cell state. **e**, Distribution of interchromosomal distances in each cell state. **f**, log_2_(FC) of mean interloci distances for AdenomaR, AdenomaY and AdenomaG relative to AT2 cells. Yellow lines highlight the boundaries of chromosomes. **g**, Distributions of radial scores of chr13, chr7 and chr19 in each cell state. **h**, Mean log_2_(FC) of radial scores of each chromosome, comparing each cancer state to the AT2 cell state. An asterisk indicates FDR < 0.05, two-sided Wilcoxon signed-rank test. **i**, log_2_(FC) of mean interloci distances for LUAD relative to AT2 cells. Yellow lines highlight the boundaries of chromosomes. **j**, Nuclear convex hull volume of WT AT2, AdenomaR, AdenomaY, AdenomaG and LUAD cells. *P* values of two-sided Wilcoxon rank-sum tests are shown. *n* = 3,410, 157, 689, 878 and 8,834 for WT AT2, AdenomaR, AdenomaG, AdenomaY and LUAD cells, respectively (cells with at least 10 traces). In **b**, **d**, **e** and **g**, *P* values of two-sided Wilcoxon signed-rank test comparing each cancer state to WT AT2 state with matched autosomes (**b**,**d**) or matched genomic loci (**e**,**g**) are displayed. In **b**, **d**, **e**, **g** and **j**, horizontal lines of each box represent the 75th percentile, median and 25th percentile. Whiskers indicate nonoutlier maximum and nonoutlier minimum, with outlier thresholds at 1.5× interquartile range beyond box limits. Source data

To further characterize 3D genome evolution during LUAD progression, we quantified several additional structural features of chromosomes across cell states, including: (1) long-range intermixing (calculated using a demixing score metric benchmarked using active and inactive X-chromosome folding conformations in human cells^11^) (Fig. 3a), (2) A–B compartment polarization (quantified using a polarization index metric^11^), (3) interchromosomal distance and (4) radial localization of each genomic locus in the nucleus (Methods). *Reduced* long-range intermixing (Fig. 3b,c) and *increased* A–B compartment polarization (Fig. 3d) were seen for all adenoma cell states compared to AT2 cells. Moreover, chromosomes in adenoma states were more spatially associated (reduced interchromosomal distance; Fig. 3e,f), with a subset (for example, chromosome 7, chromosome 13 and chromosome 19) showing consistent changes in radial distribution within the nucleus compared to AT2 cells (Fig. 3g,h). Similar to chromosome compaction, these phenotypes largely recovered in LUAD cells (Figs. 2 and 3). Notably, interchromosomal distances in LUAD largely surpassed those of AT2 cells (Fig. 3e,i), leading to an expected larger overall nuclear volume in LUAD than in AT2 and adenoma cells (Fig. 3j). Together, these data demonstrated stereotypical, nonmonotonic and stage-specific 3D genome conformations during lung cancer progression, which could represent a structural bottleneck for tumor development.

**Extended Data Fig. 4:**
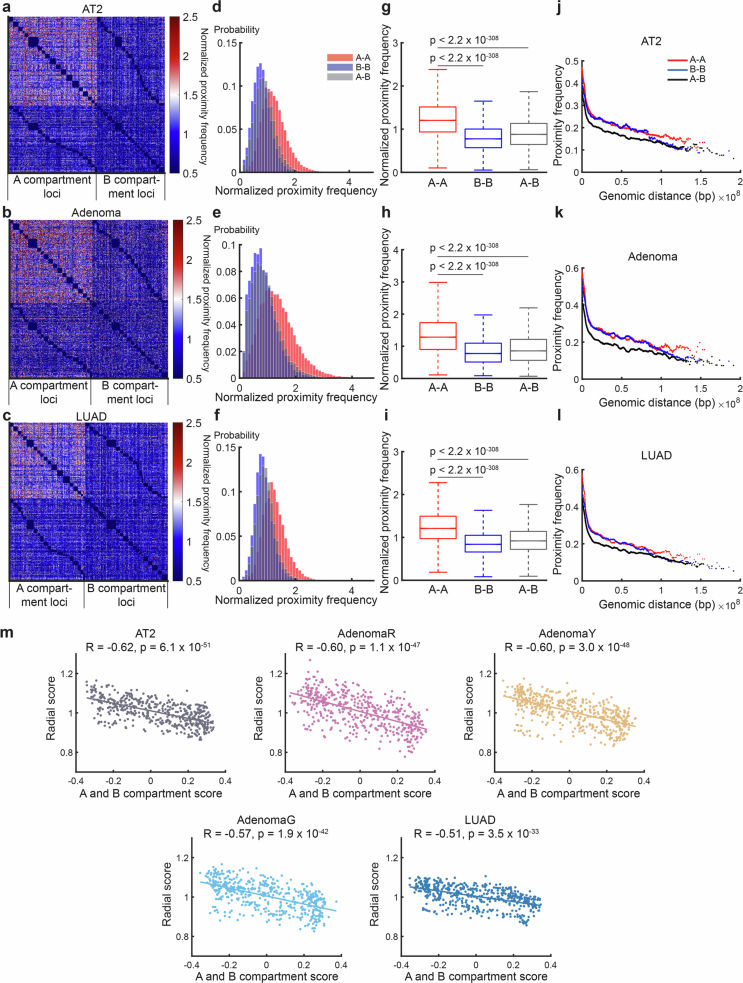
Cancer state-independent 3D genome features during lung cancer progression. **a**–**c**, Normalized *trans*-chromosomal proximity frequency between genomic loci in AT2 (**a**), adenoma (**b**) and LUAD (**c**) cells. The proximity frequency between each pair of *trans*-chromosomal genomic regions were normalized to the mean proximity frequency of all loci pairs of the two corresponding chromosomes. A cutoff distance of 800 nm was used for defining proximity. The genomic regions were re-ordered so that A compartment loci were grouped separately from B compartment loci. **d**–**f**, Distribution of normalized *trans*-chromosomal proximity frequencies of pairs of A loci (A–A), pairs of B loci (B–B) and pairs of A and B loci (A–B) in AT2 (**d**), adenoma (**e**) and LUAD (**f**) cells. **g**–**i**, Normalized *trans*-chromosomal proximity frequencies of A–A, B–B and A–B loci pairs in AT2 (**g**), adenoma (**h**) and LUAD (**i**) cells. The horizontal lines of each box from top to bottom represent the 75th percentile, the median and the 25th percentile. Whiskers extend to the nonoutlier maximum and nonoutlier minimum. Outliers are defined as values at least 1.5 times interquartile range away from the top or bottom of the box. *P* values from two-sided Wilcoxon rank-sum tests are shown. **j**–**l**, The proximity frequency between each pair of *cis*-chromosomal genomic regions as a function of their genomic distances in AT2 (**j**), adenoma (**k**) and LUAD (**l**) cells. **m**, Radial scores versus A–B compartment scores of genomic loci in the five cell states. The lines are fitted linear regression lines. Pearson correlation coefficients (*R*) and *P* values from two-sided *t*-tests are shown. *n* = 4,806, 791, 1,603, 1,941 and 17,711 for WT AT2, AdenomaR, AdenomaY, AdenomaG and LUAD cells, respectively. Cell numbers are the same in all panels. Source data

Besides the chromatin conformation alterations in cancer evolution, we also identified several cancer state-independent 3D genomic features. For example, spatial proximity frequencies were the highest for *trans*-chromosomal A–A compartment interactions, followed by A–B and B–B interactions (Extended Data Fig. 4a–i). The same trend was also observed in long-range *cis*-chromosomal compartment interactions (Extended Data Fig. 4j–l). Furthermore, we identified a consistent negative correlation between radial scores and A–B compartment scores (Extended Data Fig. 4m). These observations align with previously reported findings in mouse fetal livers and human lung fibroblasts^18,24^, arguing that they may represent general principles of 3D genome organization independent of cell state, cell type and even mammalian species.

**Extended Data Fig. 5:**
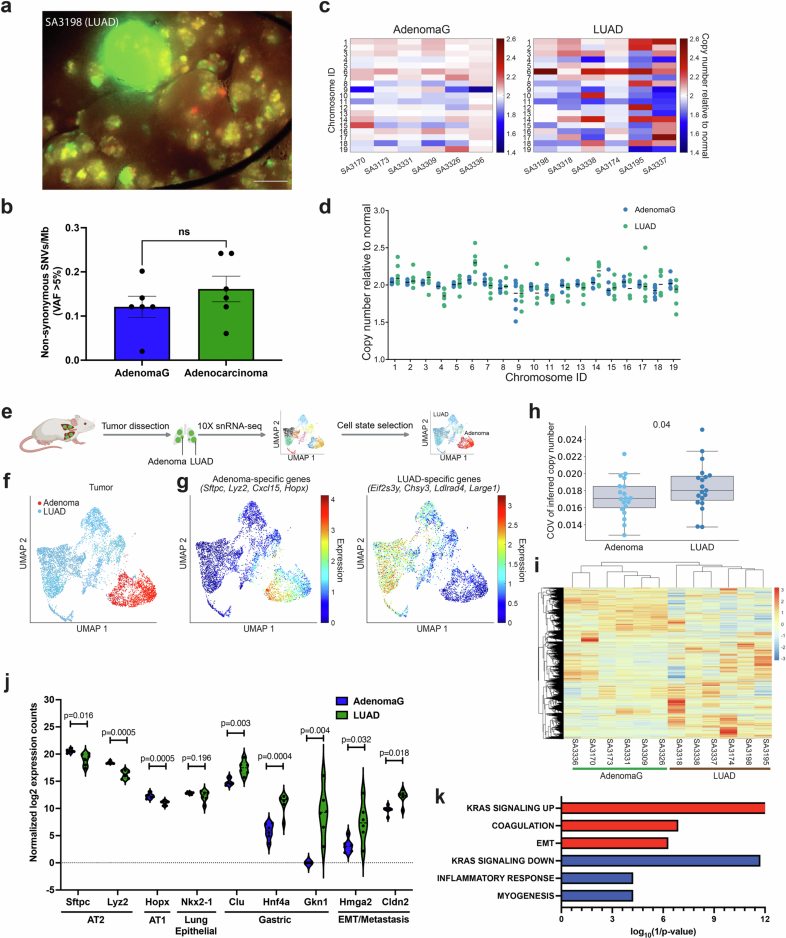
Exome and RNA sequencing analyses of lung tumors from *K-MADM-Trp53* mice. **a**, Representative large GFP+ (green) tumor for whole-exome and RNA sequencing. Scale bar = 2.5 mm. Image is representative of 6 mice. **b**, Frequency of nonsynonymous single nucleotide variants (SNVs) per megabase (Mb) with variant allele fraction (VAF) > 5% in AdenomaG and LUAD. **c**,**d**, Heatmap (**c**) and distribution (**d**) of mean copy number of each autosome across AdenomaG and LUAD (*n* = 6 tumors per group). Black lines (**d**) represent the median. **e**, Schematic illustration of snRNA-seq in *K-MADM-Trp53* mice. The schematic in panel **e** was created with BioRender.com. **f**,**g**, UMAP of single-cell gene expression profiles in *K-MADM-Trp53* lung tumors (*n* = 6,300 cells). Different cell-type clusters were labeled with different colors (**f**) based on gene set enrichment patterns of adenoma (*n* = 1,631) and LUAD (*n* = 4,669)-specific genes (**g**). **h**, Distribution of coefficient of variation of mean inferred copy numbers of all genes on each autosome (*n* = 19 autosomes) in adenoma and LUAD. *P* value from two-sided Wilcoxon signed-rank test. Horizontal lines of each box represent the 75th percentile, median and 25th percentile. Whiskers indicate nonoutlier maximum and nonoutlier minimum, with outlier thresholds at 1.5 times interquartile range beyond box limits. **i**,**j**, Unsupervised hierarchical clustering of all expressed genes (**i**) segregates dissected green tumors into two clusters (*n* = 6 tumors per cluster) identified as AdenomaG and LUAD based on known histologic progression markers (ref. ^43^; **j**). Row-normalized expression counts are shown in heatmap (**i**). Horizontal lines in each violin in **j** represent the 75th percentile, median and 25th percentile. *P* values were from two-sided Wilcoxon rank-sum tests. **k**, Gene set enrichment analysis using MSigDB Hallmarks (H1) shows top enriched gene sets for upregulated (red, log_2_ fold change > 2 and FDR < 0.05) and downregulated (blue, log_2_ fold change <−1 and FDR < 0.05) genes in LUAD over AdenomaG. *P* values were from one-sided hypergeometric tests. Source data

To determine whether the nonmonotonic 3D genome changes are driven by genetic alterations, we performed whole-exome sequencing and analyzed single nucleotide variants (SNVs) and copy number variants (CNVs) in isolated GFP+ *Kras*^*G12D*^/*Trp53*^*−/−*^ adenomas and LUAD from *K-MADM-Trp53* mice (Supplementary Table 4 and Extended Data Fig. 5a). Few SNVs were observed in both adenoma and LUAD tumors, none of which resulted in protein-altering mutations in known cancer driver genes^31^ (Extended Data Fig. 5b), as this has been previously described in the conventional *Kras*^*LSL-G12D/WT*^*; Trp53*^*flox/flox*^ (KP) LUAD model^32^. Furthermore, adenoma cells did not harbor recurrent CNVs (Extended Data Fig. 5c,d), suggesting that the observed phenotypic convergence in chromatin compaction (Fig. 2i,j) was not triggered by additional genetic evolution beyond oncogenic Kras expression as a tumor-initiating event. In contrast, a subset of LUAD tumors exhibited gains of chromosome 6 and losses of chromosomes 4, 9 and 11 (Extended Data Fig. 5c,d), also consistent with the KP LUAD model^32^. As an orthogonal approach, single-cell CNVs inferred from single-nucleus RNA sequencing (snRNA-seq) of adenoma and LUAD (Extended Data Fig. 5e–g) showed increased copy number heterogeneity in LUAD in comparison to adenoma (Extended Data Fig. 5h). These copy number alterations may partially explain the divergent recovery in heterogeneity and decompaction observed in LUAD cells relative to AT2 cells. However, the lack of systematic gains or losses across most chromosomes and tumors suggests that CNVs cannot account for the convergent global chromatin structure differences between adenoma and LUAD. Collectively, these data argue that the observed 3D genome alterations are not merely a surrogate of mutational evolution in this model.

### Conserved 3D genome reorganization in PDAC progression

**Extended Data Fig. 6:**
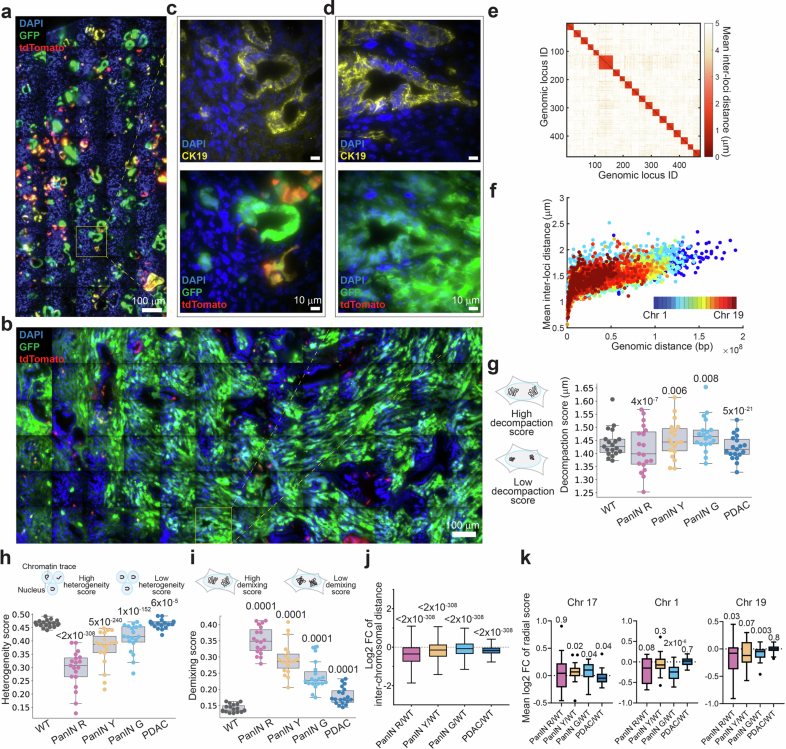
Genome-wide chromatin tracing of pancreatic ductal adenocarcinoma progression. **a**,**b**, Whole-section fluorescence images of a *K-MADM-Trp53* pancreas with PanINs (**a**) and PDAC (**b**). **c**,**d**, Immunofluorescence staining (top) of the duct cell marker CK19 and DAPI staining in a field of view, each in **a** and **b**. Fluorescent protein imaging (bottom) of GFP, tdTomato and DAPI staining in the corresponding fields. Images are from two independent experiments. The images are maximum-intensity z-projections from 10-μm z-stacks. **e**, Matrix of mean interloci distances between all genomic loci in normal duct cells. *n* = 1,529 cells. **f**, Mean interloci spatial distance versus genomic distance for all pairs of genomic loci on each autosome in duct cells. Different pseudocolors represent different autosomes. *n* = 6,039 intrachromosomal interloci pairs. *n* = 1,529 cells. **g**–**i**, Distributions of the decompaction, heterogeneity and demixing scores of each autosome (*n* = 19 autosomes) in each pancreatic cell state. *n* = 1,529, 123, 189, 361 and 475 for normal duct, PanIN R, PanIN Y, PanIN G and PDAC cells, respectively. Cell numbers of each cell state are identical in **g**–**k**. **j**, log_2_ fold change of mean interchromosomal distances, comparing each cancer state to normal duct cells. **k**, Distribution of the mean log_2_ fold change of radial scores of chr17, chr1 and chr19, comparing each cancer state to normal duct cells. *P* values of two-sided Wilcoxon signed-rank tests comparing each cancer state to WT duct cells with matched genomic loci (**g**, **h**, **j** and **k**) or matched autosomes (**i**) are shown. In **g**–**k**, the horizontal lines of each box represent the 75th percentile, median and 25th percentile. Whiskers extend to the nonoutlier maximum and nonoutlier minimum. Outliers are defined as values away from the top or bottom of the box by more than 1.5 times interquartile range. Source data

To study whether the global 3D genome changes in LUAD progression are conserved in another Kras-driven cancer type, we leveraged an analogous *K-MADM-Trp53* model of PDAC, which directs Cre recombinase activity to the pancreas using a *Pdx1-cre* transgene and recapitulates major genetic and histologic events in human PDAC progression^30,33,34^. We performed genome-wide chromatin tracing targeting the same 473 genomic loci combined with cytokeratin 19 (a marker for normal and malignant duct cells) immunofluorescence, GFP, tdTomato and DAPI imaging in the same single cells. We analyzed 2,677 cells across PDAC progression stages, including normal duct cells, preinvasive pancreatic intraepithelial neoplasia (PanIN) cells (R, Y, G) and invasive PDAC cells (Extended Data Fig. 6a–d). Mean interloci distances showed the expected chromosome territory features and genomic distance scaling (Extended Data Fig. 6e,f), indicating high-quality traces. Analyses of key chromatin folding features largely recapitulated findings in LUAD progression, including (1) intrachromosomal compaction, (2) intrachromosomal heterogeneity, (3) long-range intermixing, (4) interchromosomal distance and (5) radial localization of genomic loci in the nucleus (Extended Data Fig. 6g–k). When comparing PanIN to WT duct cells, we observed that chromosomes largely exhibited more compact folding (especially in PanIN R), decreased heterogeneity, reduced long-range intermixing, decreased interchromosomal distances and changes in radial distribution for a subset of chromosomes (for example, chromosome 1, chromosome 17 and chromosome 19). These phenotypes at least partially recovered upon progression to PDAC. Thus, stereotypical, nonmonotonic and stage-specific changes in 3D genome conformations seen in PDAC progression mirrored those in LUAD, arguing that the 3D genome changes were not limited to a single Kras-driven cancer type.

### Single-cell 3D genome distinguishes cancer cell states

**Fig. 4:**
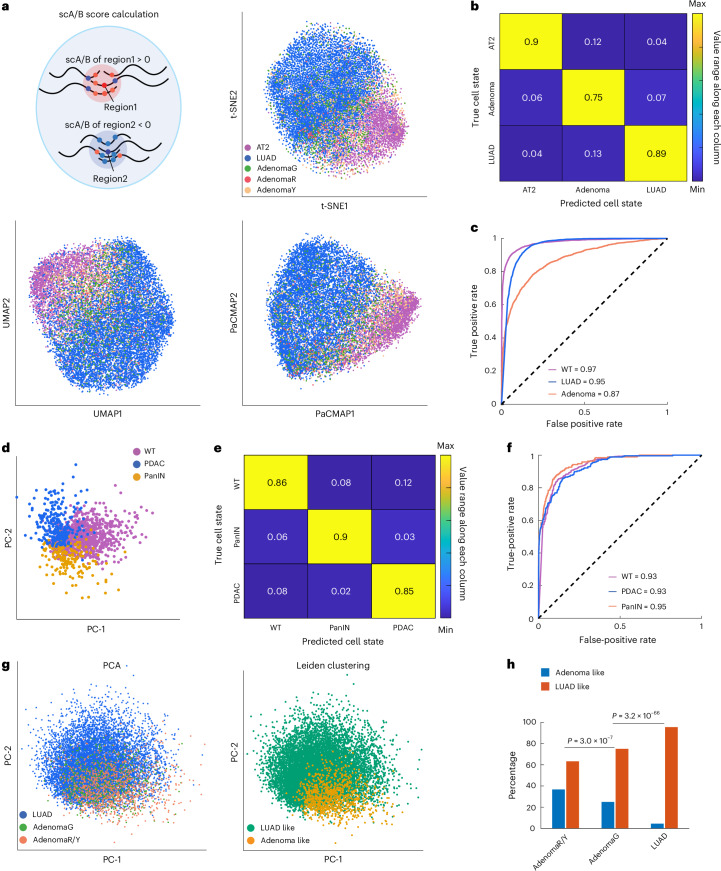
The single-cell 3D genome distinguishes and encodes cancer progression states. **a**, Cartoon illustration of scA/B score calculation (top-left). t-SNE (top-right), UMAP (bottom-left) and PaCMAP (bottom-right) plots of single-cell 3D genome conformations. *n* = 3,410, 157, 689, 878 and 8,834 for WT AT2, AdenomaR, AdenomaG, AdenomaY and LUAD cells, respectively. Cell numbers of each cell state are identical in **a**–**c**, **g** and **h**. **b**, Confusion matrix of supervised machine learning in mouse lung cells. The number in each matrix element represents the precision in each predicted state. **c**, ROC curves of the machine learning model in mouse lung cells. The AUC values are shown. **d**, PCA plot of single-cell 3D genome conformations. *n* = 1,103, 191 and 268 for normal duct, PanIN and PDAC cells, respectively. Cell numbers of each cell state are identical in **d**–**f**. **e**, Confusion matrix of supervised machine learning in mouse pancreas cells. The number in each matrix element represents the precision in each predicted state. **f**, ROC curves of the machine learning model in mouse pancreas cells. The AUC values are shown. **g**, PCA plot of single-cell 3D genome conformations of adenoma and LUAD cells (left). Leiden clustering separates Adenoma-like and LUAD-like clusters (right). **h**, Percentages of cells with adenoma-like or LUAD-like 3D genome conformations in **g**, in each of the AdenomaR/AdenomaY, AdenomaG and LUAD states. The adenoma-like or LUAD-like conformation state for each cell is assigned based on a Leiden clustering approach. *P* values from two-sided Fisher’s exact test are shown. t-SNE, *t*-distributed stochastic neighbor embedding; UMAP, uniform manifold approximation and projection; PaCMAP, pairwise controlled manifold approximation; ROC, receiver operating characteristic; AUC, area under the curve. Source data

**Extended Data Fig. 7:**
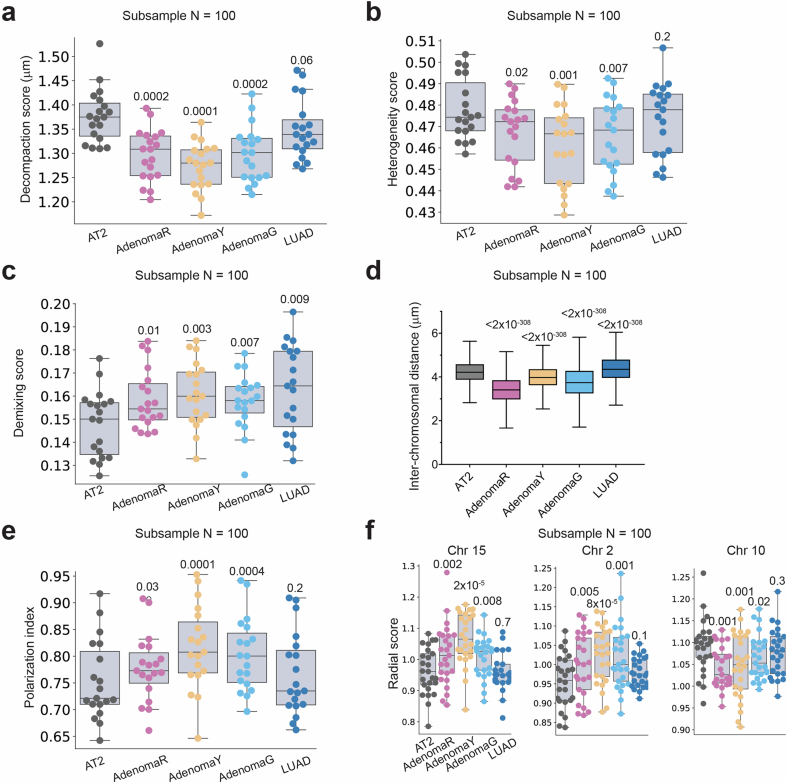
Subsampling analysis of chromatin folding changes during lung cancer progression. **a**–**c**, Distributions of the decompaction (mean interloci distance), heterogeneity (COV of interloci distance) and demixing scores of each autosome (*n* = 19) in a randomly subsampled population of 100 cells per cell state. **d**, Distribution of interchromosomal distances in a randomly subsampled population of 100 cells per cell state. **e**, Distribution of the polarization indices of A and B compartments of each autosome (*n* = 19) in a randomly subsampled population of 100 cells per cell state. **f**, Distribution of the radial scores of chr15, chr2 and chr10 in a randomly subsampled population of 100 cells per cell state. *P* values of two-sided Wilcoxon signed-rank test (**a**–**c**, **e** and **f**) and two-sided Wilcoxon rank-sum test (**d**) are displayed. In **a**–**f**, the horizontal lines of each box from top to bottom represent the 75th percentile, median and 25th percentile. Whiskers extend to the nonoutlier maximum and nonoutlier minimum. Outliers are defined as values at least 1.5 times interquartile range away from the top or bottom of the box. Source data

We next determined whether different morphologic cancer cell states can be classified with only single-cell 3D genome data. Using subsampling analysis, we first established that as few as 100 cells per cell state are sufficient to recapitulate most 3D genome changes described above (Extended Data Fig. 7). We then leveraged a ‘Trace2State’ pipeline that performs dimensionality reduction of single-cell 3D genome conformation data to visualize clusters of different morphologic cell states. We adopted a previously developed single-cell A/B (scA/B) compartment score metric for each target genomic locus (Fig. 4a)^35,36^, which is defined as the mean A–B compartment score of all its spatially adjacent loci (<1200-nm radius) and reflects the A–B compartment identity of each locus at the single-cell level. We used these scores as high-dimensional input variables to generate a 2D display using three independent dimensionality reduction methods^37–39^. Single-cell 3D genome conformations of AT2 and LUAD cells were largely distinct, whereas those of adenoma cells were clustered between them (Fig. 4a), consistent with morphologic progression. Supervised machine learning models trained with the scA/B profiles classified different cancer cell states (Methods), with a support vector machine model achieving 90% overall accuracy in predicting these states (Fig. 4b,c). Similar results were obtained for cell states during PDAC progression (Fig. 4d–f). These results support the utility of compartment-level 3D genome organization in distinguishing morphological cell states during oncogenesis at the single-cell level.

We hypothesized that the subset of *Trp53*^*−/−*^ AdenomaG cells progressing to LUAD has acquired specific 3D genome features. Indeed, AdenomaG cells often exhibited chromosome-scale structural features intermediate between those of nonprogressing AdenomaR/AdenomaY cells and LUAD cells (Figs. 2 and 3). Furthermore, 3D genome dimensionality reduction analyses showed that AdenomaR/AdenomaY cells and LUAD cells formed distinct but overlapping clusters, with AdenomaG cells dispersed within and between them (Fig. 4g). Leiden clustering^40^ segmented the low-dimensional space into adenoma-like and LUAD-like regions and revealed that more AdenomaG cells (versus other adenoma cells) adopted LUAD-like 3D conformations (Fig. 4g,h), indicating that 3D chromatin organization changes associated with LUAD precede morphologic progression.

### 3D genomics delineates prognostic and predictive biomarkers

Because AdenomaG cells were more likely to adopt LUAD-like compartment organization than nonprogressing cells (AdenomaR/AdenomaY), we examined how compartment transitions between AdenomaG and LUAD relate to gene expression. We reasoned that compartment alterations could promote stable gene activation or repression with functional consequences, due to differences in active–repressive epigenetic signatures, RNA polymerase II (Pol II) and transcription factor densities in these compartments^41^. Therefore, we postulated that genes with increased expression in genomic loci with increased scA/B scores in LUAD cells are important for LUAD progression, which we termed candidate progression drivers (CPDs). We further hypothesized that these CPDs associated with compartment changes are more homogenously expressed than genes upregulated by compartment-independent mechanisms and—similar to genes with diminished intratumoral expression heterogeneity in human lung cancer^42^—more likely to undergo positive selection during tumor progression.

**Fig. 5:**
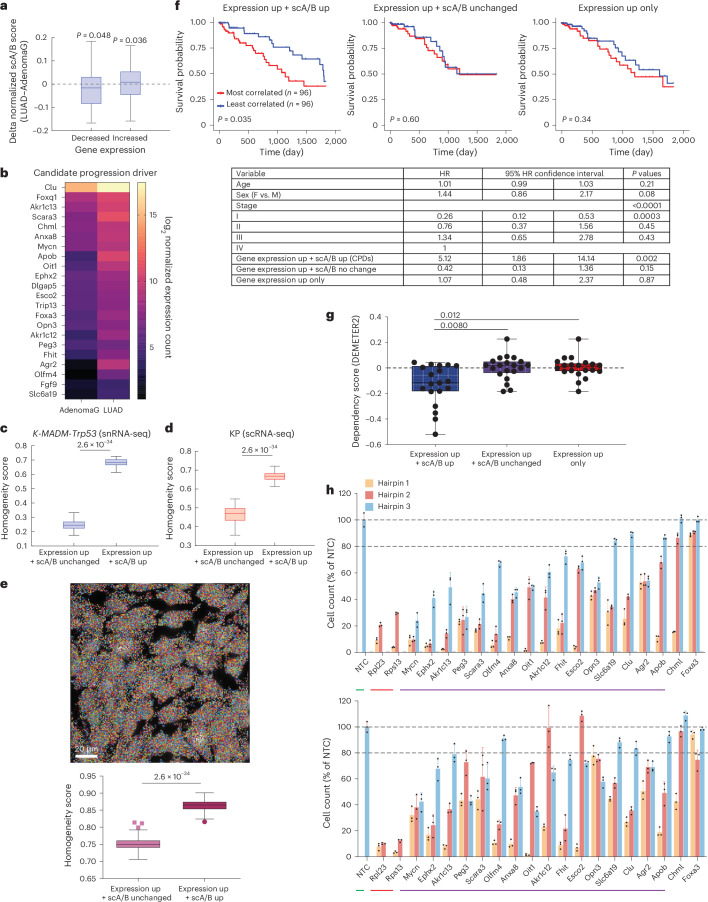
Single-cell 3D genomics nominates prognostic genes and genetic dependencies. **a**, Normalized scA/B score changes of genes with decreased (*n* = 94 genes) or increased (*n* = 120 genes) expression from AdenomaG to LUAD. *P* values were from one-sided one-sample *t* tests. **b**, Heatmap of CPD gene expression comparing AdenomaG and LUAD. **c**,**d**, Gene expression homogeneity of LUAD cells in the *K-MADM-Trp53* (**c**) and *Kras*^*LSL-G12D/WT*^*; Trp53*^*flox/flox*^ (KP) (**d**) models comparing CPDs (expression up + scA/B up) with control genes (expression up + scA/B unchanged). *P* values were from two-sided Wilcoxon rank-sum tests. **e**, Decoded RNA MERFISH image (top) with RNA molecules pseudo-colored by gene identities in the SA6082inf LUAD cell line. Gene expression homogeneity (bottom) across single cells comparing CPDs with control genes. *P* values were from two-sided Wilcoxon rank-sum tests. **f**, Kaplan–Meier survival curves (top) comparing TCGA LUAD patients with gene expression profiles most or least (top versus bottom 20%) correlated with CPDs and controls (expression up + scA/B unchanged; expression up only). A total of 21 genes are included per panel. *n* = 96 tumors per group. *P* values were from two-sided log-rank tests. A multivariate Cox regression model (bottom) analyzing CPD and control signatures in predicting patient survival after controlling for clinical covariates across the TCGA LUAD dataset (*n* = 478). **g**, Dependency scores (DEMETER2) of LUAD cell lines (*n* = 57) comparing CPDs and controls (expression up + scA/B unchanged or expression up only). *n* = 19–20 genes per group with available RNAi screen data in Cancer Dependency Map. Lower score = more dependent. *P* values were from the Kruskal–Wallis test with Dunn’s post hoc test. **h**, Cell viability (mean ± s.d., normalized to NTC, *n* = 3 replicates per hairpin, 3 hairpins per gene) of arrayed RNAi screen targeting CPDs in KP (top) and SA6082inf (bottom) cells. NTC (green), positive controls (red) and CPDs with substantial phenotypes (≥2 hairpins with <80% of NTC cell count, purple) are indicated. In **a**, **c**–**e** and **g**, horizontal lines of each box represent the 75th percentile, median and 25th percentile. Whiskers in **a** and **c**–**e** indicate nonoutlier maximum and nonoutlier minimum, with outlier thresholds at 1.5× interquartile range beyond box limits. Whiskers in **g** indicate maximum and minimum. NTC, nontargeting control. Source data

To link 3D genome alterations with gene expression during LUAD progression, we first performed bulk RNA-seq on GFP+ tumors of *K-MADM-Trp53* mice (Extended Data Fig. 5a and Supplementary Table 5). Unsupervised hierarchical clustering revealed two distinct tumor groups (Extended Data Fig. 5i) corresponding to AdenomaG and LUAD cells based on expected changes in gene expression with progression (Extended Data Fig. 5j,k)^43^. Genes upregulated or downregulated in LUAD (versus AdenomaG) showed increased or decreased scA/B scores, respectively (Fig. 5a). On the contrary, no substantial associations were identified between gene expression changes and scA/B score changes by randomly subsampling two groups of cells (each with equal size as AdenomaG or LUAD) for scA/B score changes (*P* = 0.46, downregulated genes; *P* = 0.66, upregulated genes). We next developed a ‘Trace2Biomarker’ pipeline to call CPDs (genes with concurrently increased scA/B score and gene expression in LUAD, Fig. 5b; Methods). To test the hypothesis that A–B compartment organization contributes to more stable gene expression regulation, we quantified gene expression heterogeneity/homogeneity in LUAD cells derived from *K-MADM-Trp53* mice analyzed by snRNA-seq (Extended Data Fig. 5e–g). Using a cosine similarity metric to quantify gene expression homogeneity across single cells, CPD genes showed substantially higher expression homogeneity than top upregulated genes in regions with unchanged scA/B scores (Fig. 5c). We validated this finding in an independent scRNA-seq dataset from the conventional KP LUAD model^43^ (Fig. 5d). Strikingly, RNA MERFISH established that CPD gene expression homogeneity was maintained even in a LUAD cell line isolated from the *K-MADM-Trp53* mouse model (SA6082inf) (Fig. 5e), highlighting the potential functional importance of CPDs. Collectively, these observations suggest that genes associated with compartment changes during tumor evolution are more homogeneously regulated, leading to a more stable expression of potential driver genes than compartment-independent regulatory mechanisms.

To investigate the clinical relevance of CPD genes, we scored individual LUAD patient samples from The Cancer Genome Atlas (TCGA)^25^ for their correlation with the CPD gene signature. Patients in the top correlated quintile (*n* = 96) exhibited substantially worse survival than patients in the most anticorrelated quintile (*n* = 96; Fig. 5f). In contrast, top differentially expressed genes in genomic regions with unchanged scA/B scores were poor predictors of survival (Fig. 5f). A multivariate Cox regression analysis controlling for available clinical covariates (age, sex and stage) across the entire TCGA LUAD dataset (*n* = 478) confirmed that the CPD gene signature was independently associated with worse survival (hazard ratio (HR) = 5.12, *P* = 0.002; Fig. 5f), whereas the top upregulated genes in genomic regions with unchanged scA/B scores or irrespective of scA/B score were not. LUAD cell lines from the Cancer Dependency Map^44^ also showed greater dependency for CPD genes than for the control gene sets (Fig. 5g). Finally, we performed an arrayed lentiviral RNAi screen targeting 18 CPD genes with short hairpin RNAs (shRNAs, *n* = 3 per gene; Supplementary Table 6) in two primary LUAD mouse cell lines (SA6082inf from the *K-MADM-Trp53* model and 31671 from the conventional KP model^45^). Knockdown of 16 of 18 (89%) CPD genes reduced viability in both cell lines (Fig. 5h)—including five targets not previously implicated in lung cancer (*Ephx2*, *Peg3*, *Apob*, *Oit1* and *Slc6a19*; Supplementary Table 7). In contrast, only 6.4% random genes would affect cell proliferation in the Cancer Dependency Map^46–49^ (Supplementary Note). These results suggest that combining 3D genome organization and gene expression data enhances both prognostic (survival) and predictive (genetic dependency) power beyond gene expression alone.

### Enhancer–promoter (E–P) contacts in CPDs

**Extended Data Fig. 8:**
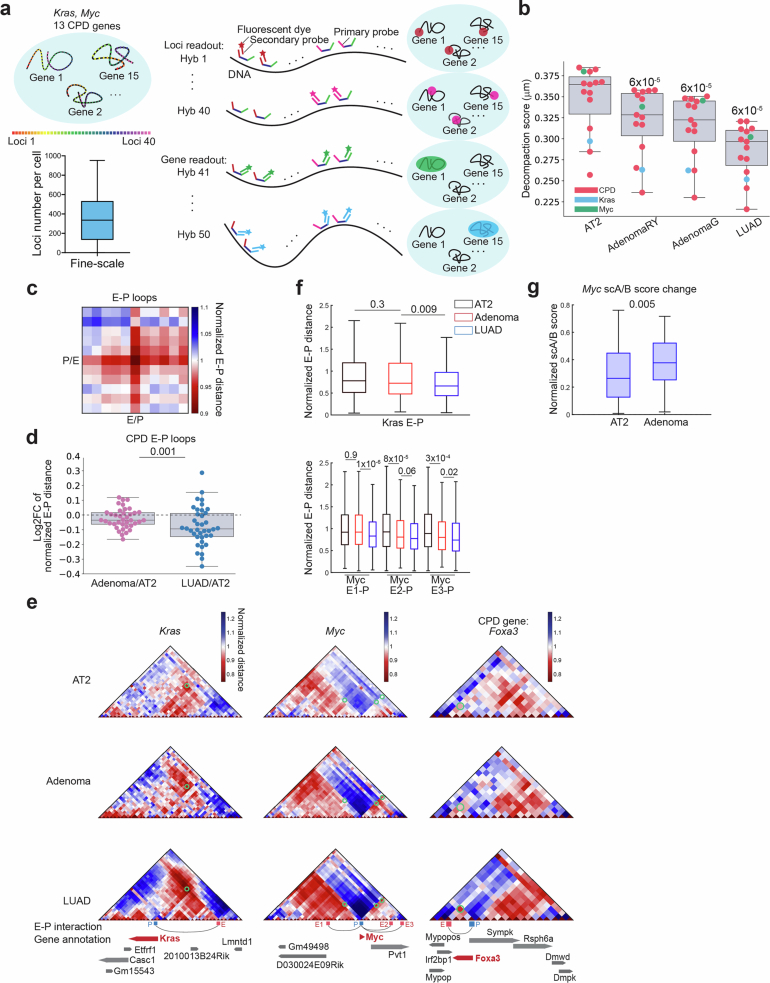
Fine-scale tracing of candidate drivers of cancer progression. **a**, Schematic illustration of high-resolution chromatin tracing targeting the *cis*-regulatory regions of 15 genes, including CPD, *Kras* and *Myc*. Distribution of detected loci number per cell is displayed. *n* = 12,661 cells combining the five cell states. **b**, Distribution of the decompaction (mean interloci distance) scores of all target genes. *n* = 2,328, 654, 558 and 9,121 for normal AT2, AdenomaR/Y, AdenomaG and LUAD cells, respectively. Cell numbers in each cell state are identical in **b**–**f**. *P* values of two-sided Wilcoxon signed-rank tests are displayed. **c**, Pileup heatmap of normalized E**–**P distances centered around each E**–**P loop. E**–**P loops of CPD genes are called in LUAD cells. **d**, Distribution of the log_2_ fold change of the normalized distances between promoter and putative enhancers of CPD genes, comparing each cancer state to AT2 cells. *P* value of two-sided Wilcoxon signed-rank test is displayed. **e**, Normalized interloci distance matrices of the target genomic regions surrounding the *Kras*, *Myc* and *Foxa3* (CPD) genes in AT2, adenoma and LUAD cell states. The green circles designate putative enhancer–promoter contacts. Putative enhancer and gene annotation tracks are aligned to the target regions. **f**, Distribution of the normalized distances between promoter and putative enhancers of the *Kras* and *Myc* genes. *P* values of two-sided Wilcoxon rank-sum tests are displayed. **g**, Distribution of the normalized scA/B scores of *Myc* gene-containing target genomic region in AT2 and adenoma cells. *P* value of two-sided Wilcoxon rank-sum test is displayed. In **a**, **b**, **d**, **f** and **g**, the horizontal lines of each box from top to bottom represent the 75th percentile, median and 25th percentile. Whiskers extend to the nonoutlier maximum and nonoutlier minimum. Outliers are defined as values at least 1.5 times interquartile range away from the top or bottom of the box. Source data

To assess local chromatin structure changes of CPD genes at higher resolution during LUAD progression, we performed fine-scale chromatin tracing of the *cis*-regulatory regions of 15 genes, including CPDs and the known oncogenes *Kras* and *Myc* (Extended Data Fig. 8a). For each gene, we targeted 40 consecutive 5–20-kb loci (20 loci for *Foxa3* due to short genomic length) spanning the promoter and candidate enhancers (Extended Data Fig. 8a) and profiled a total of 12,661 AT2, AdenomaR, AdenomaY, AdenomaG and LUAD cells with a median of 336 loci detected per cell (Extended Data Fig. 8a). The kilobase-resolution chromatin folding became increasingly more compact during progression from AT2 to adenoma and then to LUAD (Extended Data Fig. 8b), suggesting that the trend of chromatin compaction from adenoma to LUAD depends on the length scale, as previously reported for chromatin compaction in other contexts^50–52^. To identify E–P interactions, we accounted for the confounding gene-scale compaction and analyzed normalized interloci distances between putative active enhancers and promoters (Supplementary Note and Extended Data Fig. 8c). Most E–P loops for CPD genes exhibited increased interactions during progression from adenoma to LUAD, concordant with increased gene expression (Extended Data Fig. 8d,e). *Kras* showed increased E–P looping in LUAD (Extended Data Fig. 8e,f), suggesting an alternative mechanism for enhancing Kras expression in the adenoma-to-LUAD transition beyond amplification^32^. Similarly, *Myc*—basal expression of which is required for *Kras*-driven lung tumor initiation^53^ and overexpression of which is sufficient to induce lung tumorigenesis^54^—showed increased looping between its promoter and two putative downstream enhancers (Extended Data Fig. 8e,f) and increased scA/B scores (Extended Data Fig. 8g) in the AT2-to-adenoma transition, concordant with increased expression. Collectively, these data suggest that both local looping and larger-scale compartmental alterations may enhance driver gene expression.

### Rnf2 regulates 3D genome through a noncanonical function

**Extended Data Fig. 9:**
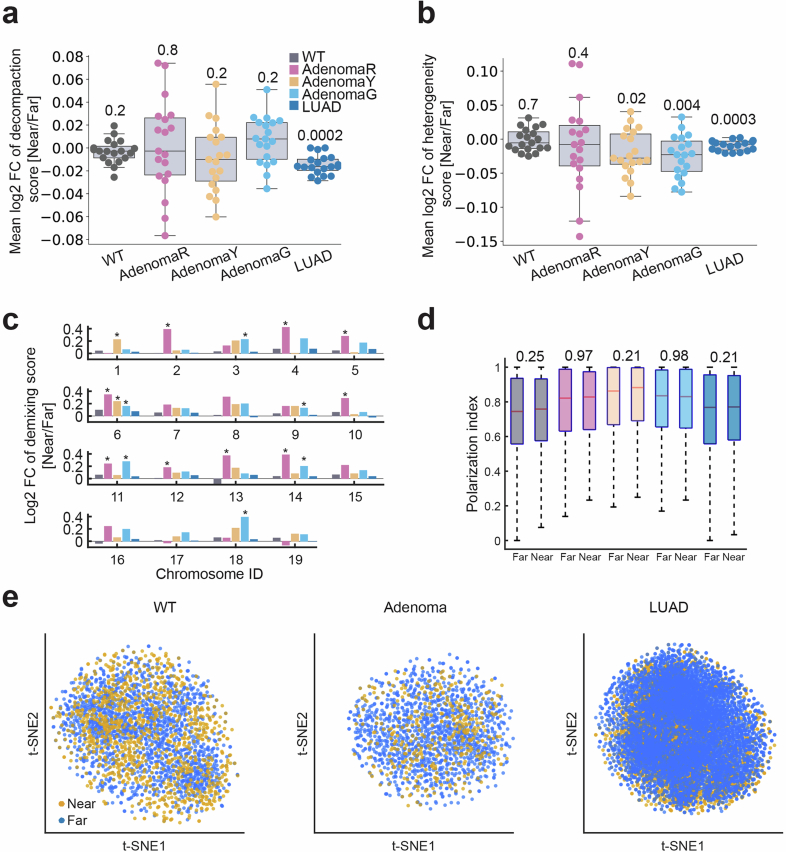
3D genome organization in cancer cells is largely independent of spatial proximity to immune cells. **a**–**c**, Mean log_2_ fold change of decompaction (**a**), heterogeneity (**b**) and demixing (**c**) scores of each chromosome, comparing AT2/cancer cells near (less than 10 μm) versus far (more than 10 μm) from CD45+ immune cells. The *P* values (**a**,**b**) or FDR (**c**) with significance (<0.05) from two-sided Wilcoxon signed-rank test (**a**,**b**) or two-sided Levene’s test (**c**) are displayed. **d**, Distribution of polarization indices of A–B compartments in AT2/cancer cells near or far from immune cells. Two-sided Wilcoxon rank-sum test yielded no significant *P* values (*P* < 0.05). **e**, t-SNE plots of single-cell 3D chromatin conformations in AT2, adenoma and LUAD cells show no distinct clusters based on spatial proximity to immune cells. In **a**, **b** and **d**, the horizontal lines of each box from top to bottom represent the 75th percentile, median and 25th percentile. Whiskers extend to the nonoutlier maximum and nonoutlier minimum. Outliers are defined as values at least 1.5 times interquartile range away from the top or bottom of the box. In **a**-**d**, *n* = 3,806, 758, 1,563, 1,882 and 17,711 for WT AT2, AdenomaR, AdenomaY, AdenomaG and LUAD cells, respectively (excluding early AT2 and adenoma replicates without CD45 labeling). In **e**, *n* = 2,719, 1,710 and 8,834 for WT AT2, Adenoma and LUAD cells, respectively (cells with at least 10 traces). Source data

To identify potential regulators of 3D genome architecture in LUAD progression, we first assessed whether neighboring immune cells in the tumor microenvironment affected 3D genome organization and found little influence. Analysis of AT2/cancer cells delineated by proximity to CD45+ cells showed negligible differences in chromatin compaction, conformation heterogeneity, demixing, and A–B compartment polarization (Extended Data Fig. 9a–d). Dimensionality reduction further revealed that AT2/cancer cells adjacent to and distant from immune cells contained similar spectra of single-cell 3D genome conformations (Extended Data Fig. 9e). These findings suggest that changes in 3D genome organization during LUAD progression are likely independent of immune cell interactions.

**Fig. 6:**
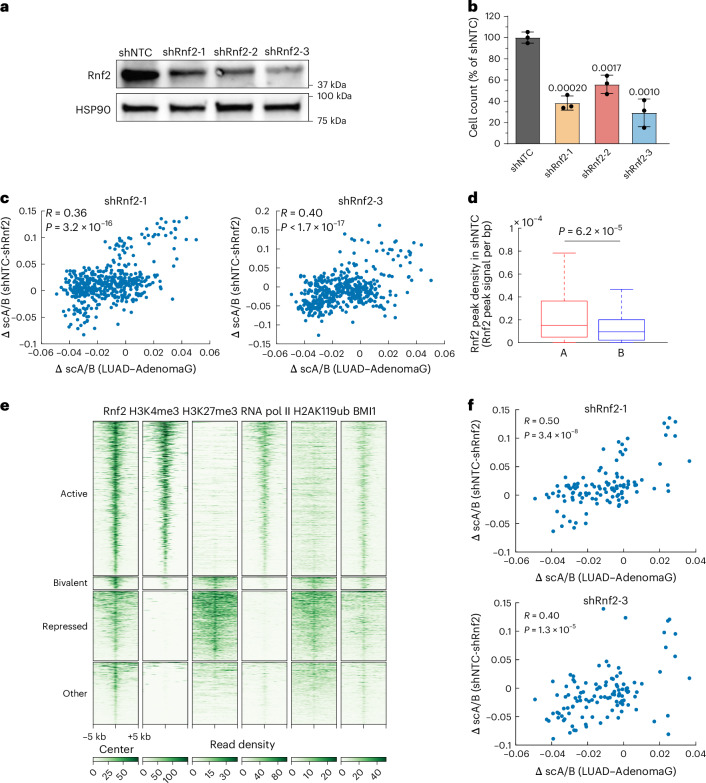
Rnf2 partially regulates 3D genome organization changes during the adenoma-to-LUAD transition. **a**, Western blot analysis of Rnf2 protein levels following Rnf2 knockdown with three independent hairpins in the KP cell line. Control cells were constructed with an NTC shRNA sequence (shNTC). HSP90 is loading control. **b**, Cell viability (mean ± s.d., normalized to mean of shNTC) following knockdown with three Rnf2 shRNAs, *n* = 3 replicates per hairpin. *P* values from two-sided two-sample *t* tests are shown. **c**, scA/B score changes from shRnf2 to shNTC versus those from AdenomaG to LUAD show a substantial positive correlation, *n* = 473 target genomic regions. Spearman correlation coefficients and *P* values from two-sided *t* tests are shown. **d**, Rnf2 peak densities in A (*n* = 209 regions) and B (*n* = 264 regions) compartments in shNTC. The horizontal lines of each box from top to bottom represent the 75th percentile, median and 25th percentile. Whiskers extend to the nonoutlier maximum and nonoutlier minimum. Outliers are defined as values at least 1.5× interquartile range away from the top or bottom of the box. *P* value of two-sided Wilcoxon rank-sum test is shown. **e**, CUT&RUN read density heatmaps of Rnf2, H3K4me3, H3K27me3, RNA polymerase II with phosphorylated S5 modification, mono-ubiquitination of lysine 119 of histone H2A (H2AK119ub) and BMI1 in shNTC KP cells. Rnf2 peak regions (−5 kb, +5 kb) of all target genomic regions are shown and are categorized as active (H3K4me3^+^, H3K27me3^−^), bivalent (H3K4me3^+^, H3K27me3^+^), repressed (H3K4me3^−^, H3K27me3^+^) or other (H3K4me3^−^, H3K27me3^−^) based on chromatin marks. **f**, scA/B score changes from shRnf2 to shNTC versus those from AdenomaG to LUAD show a stronger or similarly positive correlation using target genomic regions with only active Rnf2 peaks, *n* = 113 target genomic regions. Spearman correlation coefficients and *P* values from two-sided *t* tests are shown. Source data

To identify possible cell-autonomous upstream regulators of cancer 3D genome reorganization, we developed a ‘Trace2Regulator’ pipeline (Methods) using the Binding Analysis for Regulation of Transcription (BART) algorithm^55,56^ to predict transcription factors and chromatin regulators enriched at upregulated genes in loci with increased scA/B scores in the adenoma-to-LUAD transition. Rnf2, a major component of the polycomb repressive complex 1 (PRC1) catalyzing mono-ubiquitination of lysine 119 of histone H2A (H2AK119ub)^57^, emerged as a top candidate. Canonically, PRC1 works in concert with PRC2, which deposits trimethylation of lysine 27 of histone H3 (H3K27me3) to repress transcription^58^. We knocked down Rnf2 (shRnf2) in KP LUAD cells (Fig. 6a) and observed a substantial negative effect on cell viability (Fig. 6b). scA/B score changes upon Rnf2 knockdown substantially correlated with those during LUAD progression (Fig. 6c), whereas random shuffling of the control and Rnf2 knockdown cell identities led to no correlation (Spearman *R* = −0.07, *P* = 0.1 for shRnf2-1; *R* = −0.02, *P* = 0.7 for shRnf2-3). These data suggest that Rnf2 partially regulates 3D genome reorganization in LUAD cells.

Because predicted Rnf2 binding sites coincided with increased scA/B scores, we speculated that Rnf2 may preferentially bind A compartment regions. Indeed, cleavage under targets and release using nuclease (CUT&RUN) of Rnf2 revealed higher Rnf2 peak density in compartment A regions (Fig. 6d). Although Rnf2 canonically mediates gene silencing through polycomb repression^57^, it can also noncanonically associate with epigenetically active loci^59,60^. As both polycomb-repressed and active chromatin regions tend to localize in compartment A^61,62^, we evaluated the chromatin states of Rnf2-bound regions. Specifically, we clustered Rnf2 CUT&RUN peaks in all target genomic regions with histone H3 lysine 4 trimethylation (H3K4me3) and H3K27me3 CUT&RUN profiles. Most Rnf2 peaks (51.6%) resided in active regions (H3K4me3^+^, H3K27me3^−^; Fig. 6e), and fewer (23.3% and 4.3%, respectively) were in repressed (H3K4me3^−^, H3K27me3^+^) and bivalent (H3K4me3^+^, H3K27me3^+^) regions (Fig. 6e). CUT&RUN of RNA Pol II with phosphorylated S5 confirmed the active transcriptional states of the active regions (Fig. 6e). Notably, scA/B score changes upon Rnf2 knockdown were better or similarly correlated with those in LUAD progression, if only active Rnf2-bound regions were analyzed (Fig. 6f). These results suggest that Rnf2 in active loci may regulate 3D genome reorganization associated with LUAD progression.

**Extended Data Fig. 10:**
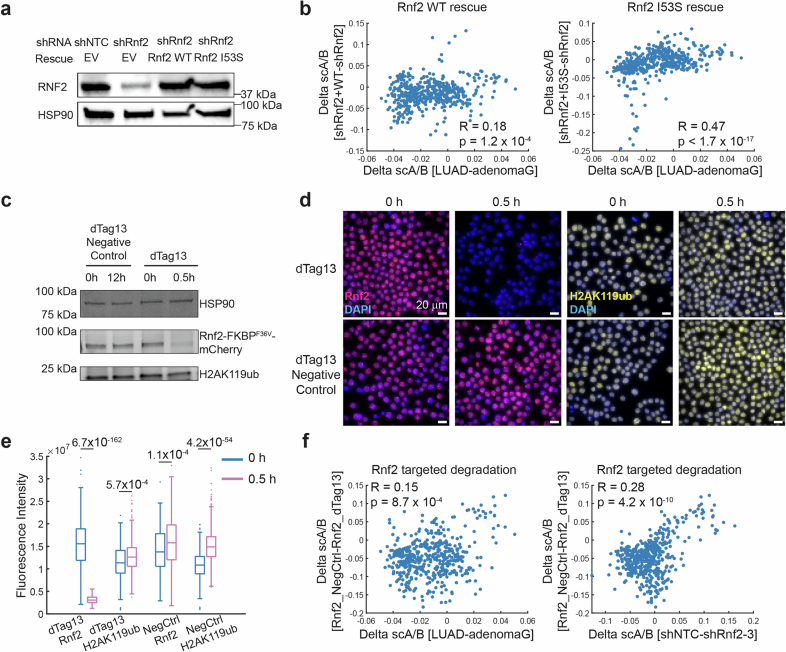
Rnf2 regulates 3D genome organization through a ubiquitin ligase-independent activity. **a**, Western blot analysis of Rnf2 protein levels following rescue of shRNA knockdown (shRnf2-3) with stable transduction of Rnf2 WT, ubiquitin ligase dead Rnf2 I53S mutant or empty vector (EV) control. HSP90 is loading control. Results were repeated in three biological replicates. **b**, scA/B score changes from shRnf2-3 to shRnf2-3 + WT Rnf2 (left) and to shRnf2-3 + I53S Rnf2 (right) versus those from AdenomaG to LUAD. *n* = 473 target genomic regions. Spearman correlation coefficients and *P* values from two-sided *t*-tests are shown. **c**, Western blot of Rnf2 and H2AK119ub in KP LUAD cells with Rnf2-dTAG after treatment with the dTAG-13 ligand or negative control ligand (a diastereomer of dTAG-13) at the designated times (0, 0.5 or 12 h). Results were repeated in three biological replicates. **d**, Immunofluorescence images of Rnf2 and H2AK119ub in KP LUAD cells with Rnf2-dTAG after treatment with dTAG-13 ligand or negative control at designated times. **e**, Quantification of the immunofluorescence intensities in **d**. *n* = 461, 534, 177, 1,040, 214, 602, 251 and 1,168 cells from left to right. The horizontal lines of each box from top to bottom represent the 75th percentile, median and 25th percentile. Whiskers extend to the nonoutlier maximum and nonoutlier minimum. Outliers are defined as values at least 1.5 times interquartile range away from the top or bottom of the box. *P* value from two-sided Wilcoxon rank-sum test is shown. **f**, scA/B score changes from Rnf2-degraded cells (dTAG-13) to Rnf2 nondegraded cells (dTAG-13 negative control) versus those from AdenomaG to LUAD and those from shRnf2 to shNTC, with *n* = 473 target genomic regions. Spearman correlation coefficients and *P* values from two-sided *t*-tests are shown. Source data

We further performed H2AK119ub CUT&RUN and found that Rnf2 peaks in active regions did not colocalize with strong H2AK119ub peaks, while another core PRC1 component, BMI1, colocalized with Rnf2 in all regions (Fig. 6e). This suggests that the canonical ubiquitin ligase function of Rnf2 may be dispensable for its role in shaping the 3D genome. Indeed, rescue experiments following Rnf2 knockdown by expressing WT Rnf2 or a catalytic-mutant Rnf2 (I53S) lacking ubiquitin ligase activity^63^ both showed scA/B score changes correlated with those during LUAD progression (Extended Data Fig. 10a,b). These results demonstrate that Rnf2 partially regulates 3D genome changes during the adenoma-to-LUAD progression through a ubiquitin ligase-independent function. To test whether Rnf2 directly reorganizes the 3D genome in LUAD cells, we used a degradation tag (dTAG) system^64^ to induce acute Rnf2 protein loss in KP cells. Rnf2 protein was rapidly degraded after 0.5 h of dTAG ligand (dTAG-13) administration but H2AK119ub levels were unchanged (Extended Data Fig. 10c–e). We found that the scA/B score changed substantially upon rapid Rnf2 degradation and these alterations correlated with those during LUAD progression or upon Rnf2 knockdown (Extended Data Fig. 10f). In contrast, random shuffling of degraded and control cell identities led to no substantial correlation (Spearman *R* = 0.04, *P* = 0.4 for LUAD progression; *R* = 0.02, *P* = 0.6 for Rnf2 knockdown). These results support a direct role for Rnf2 in 3D genome reorganization independent of its ubiquitin ligase function.

## Discussion

In this study, we generated comprehensive 3D genome atlases in physiologically relevant mouse models of Kras-driven lung and pancreatic cancer progression and introduced a generally applicable pipeline to measure and interpret biological heterogeneity of the 3D genome in complex tissues and diseases (Supplementary Fig. 1). Notably, we showed that single-cell 3D genome organization can distinguish and predict morphologic cancer states with high accuracy (Fig. 4), despite the intrinsic variability of 3D chromatin conformation even among cells of the same type^50,65–68^. We further showed that high-dimensional 3D genome data can be used to identify genes that govern prognosis and delineate cancer cell dependencies (Fig. 5). Finally, our results underscore the potential of 3D genome data to identify previously unappreciated 3D genome regulators, such as Rnf2, which appears to have a noncanonical activating function during lung tumor evolution (Fig. 6 and Extended Data Fig. 10).

Recent scRNA-seq^43^ and single-cell assay for transposase-accessible chromatin^69^ studies in Kras-driven LUAD models have revealed transitional and plastic cell states with molecular alterations that can drive tumor initiation, progression and metastasis^20,43,69^. While transcriptional heterogeneity gradually increased during LUAD progression in these models, we observed a striking nonmonotonic evolution of the 3D genome (Fig. 2), suggesting a potential 3D genome structural bottleneck. Comparison of 3D genomes across tumors and mice suggested that the increased compaction and decreased heterogeneity of chromatin conformations in adenoma cells may be due to phenotypic convergence. Although additional genetic alterations were not observed (Extended Data Fig. 5a–d), this convergence could be due to the driver oncogene *Kras*, as similar 3D genome structural changes were observed in a separate Kras-driven PDAC model (Extended Data Fig. 6). In support of a functional role of these 3D genome alterations during lung tumor evolution, combining 3D genome and transcriptomic data improved the identification of important genes (CPDs) that predict prognosis and dependency (Fig. 5). These genes are more homogenously expressed across LUAD cells both in vitro and in vivo, consistent with longitudinal data from the human tracking cancer evolution through therapy (TRACERx) lung cancer cohort demonstrating that genes with decreased intratumoral heterogeneity undergo positive selection^70^. To directly map 3D genome alterations to transcriptionally and epigenetically defined cell states, future studies should combine genome-wide chromatin tracing with spatial transcriptomics^71–74^, epigenomics^75^ and proteomics^67,76^ in the same single cancer cells in native tissues. Furthermore, as DNA FISH has been used to map structural variations in human cancer, broad application of single-cell in situ 3D genome mapping to clinical samples may hold promise for advancing cancer diagnosis, subtype classification, treatment response prediction and target discovery.

## Methods

### Ethics Statement

All animal studies were approved by the Institutional Animal Care and Use Committee of Yale University. The tumor sizes in this study did not exceed the maximal tumor size (1 cm^3^) permitted by the institutional review board.

### Mouse and cell lines

#### Mouse strains

Mice were housed in a specific-pathogen-free facility with controlled temperature, humidity and day/night cycles and maintained in a mixed background. *MADM11-GT* (013749), *MADM11-TG* (013751) and *Pdx1-cre* (014647) were obtained from the Jackson Laboratory (JAX). *Kras*^*LSL-G12D*^ (JAX, 008179) and *Trp53*^*KO*^ (JAX, 002101) mice were a gift from T. Jacks. *Kras*^*LSL-G12D*/*WT*^*; MADM11-TG, Trp53*^*KO*^/*MADM11-TG, Trp53*^*WT*^ breeder mice were generated as previously described^30^. *Kras*^*LSL-G12D*/*WT*^*; MADM11-TG, Trp53*^*KO*^/*MADM11-TG*, *Trp53*^*WT*^ were crossed with *MADM11-GT* mice (with or without *Pdx1-cre*) to generate *K-MADM-Trp53* or *Pdx1-cre; K-MADM-Trp53* experimental mice of both sexes for lung and pancreatic cancer analyses, respectively. Genotyping primers and protocols have been previously reported^30^. Large-scale chromatin tracing in lung included four WT mice (91-day male, 191-day female, 283-day male and 91-day female), four mice with adenoma (225-day male, 221-day male, 232-day female and 224-day male) and five mice with LUAD (232-day male, 487-day male, 494-day male, 221-day male and 551-day male). Large-scale chromatin tracing in pancreas included a WT mouse (59-day male) and a mouse with PanIN and PDAC tumors (46-day male). Fine-scale chromatin tracing in mouse lung included a WT mouse (191-day female), a mouse with adenoma (225-day male) and a mouse with LUAD (494-day male).

#### Lentivirus production and infection

pPGK-Cre lentiviral backbone was amplified and transfected into 293T cells using VSV-G (Addgene, 8454) and psPAX2 (Addgene, 12260) packaging vectors and TransIT-LT1 kit (MirusBio). After 48–72 h, virus was collected, filtered and ultracentrifuged. Lentivirus was resuspended in OptiMEM (Thermo Fisher Scientific) and administered intratracheally to *K-MADM-Trp53* mice at 6–10 weeks of age to generate lung tumors.

#### Cell line

The KP LUAD cell line, 31671, was a gift from N. Joshi and was derived from an autochthonous *Kras*^*LSL-G12D*/*WT*^*; Trp53*^*flox*/*flox*^ mouse administered with Adeno-Cre^77^. The *K-MADM-Trp53* LUAD cell line (SA6082inf) was derived from collagenase-based dissociation of a large GFP+ tumor dissected from a *K-MADM-Trp53* mouse. *Kras* and *Trp53* genotypes were confirmed by PCR. Both cell lines were cultured in DMEM (Corning, 10-013-CV) containing 10% (vol/vol) FBS (Gibco, 26140-079) and 1% (vol/vol) Penicillin-Streptomycin (Thermo Fisher Scientific, 15140-122) at 37 °C with 5% CO_2_. Cells were passaged whenever they reached confluency. Both cell lines were seeded onto UV-sterilized coverslips (Bioptechs, 40-mm diameter; 1.5) and allowed to grow until 60–70% confluency before primary probe hybridization. All cell lines were tested for the presence of mycoplasma using PCR (ATCC, 30-1012K).

### Probe design and synthesis

Detailed probe design and synthesis information are included in the Supplementary Note. Template probe sequences for chromatin tracing and RNA MERFISH are provided in Supplementary Table 1. Codebooks for chromatin tracing and RNA MERFISH, and genome coordinates and target region features in chromatin tracing are provided in Supplementary Table 2. PCR primers, reverse transcription primers for probe synthesis and adaptors, readout and blocking probes for sequential readout imaging are provided in Supplementary Table 3.

### Mouse *K-MADM-Trp53* lung tissue chromatin tracing experiments

#### Tissue preparation

*K-MADM-Trp53* mice were dissected at sign of respiratory distress through CO_2_ asphyxiation. Lungs were perfused with ice-cold 1× PBS, incubated in 4% (vol/vol) paraformaldehyde (Electron Microscopy Sciences, 15710-S) overnight, and cryoprotected with 30% sucrose. Tissue was embedded in Tissue-Tek optimal cutting temperature compound (OCT) and frozen on dry ice before storage at −80 °C.

#### Tissue sectioning

Frozen mouse lung blocks were sectioned at a thickness of 10 μm at −20 °C on a cryostat. The following three consecutive tissue slices were sectioned at a time: one for hematoxylin and eosin (H&E) staining, one for whole-section fluorescence imaging and one for chromatin tracing. The sections were air-dried at room temperature for 1 h and then either used directly or stored at −20 °C for months.

#### H&E staining and whole-section fluorescence imaging

H&E staining was performed by Yale Pathology Tissue Services. For whole-section fluorescence imaging, the section was mounted in mounting medium with DAPI (VectorLabs, H-1800-2). Stitched fluorescence images in 353-nm, 488-nm and 592-nm illumination channels for DAPI, GFP and tdTomato, respectively, were collected with a Plan-Apochromat ×10/0.45 M27 objective on a Zeiss Axio Imager M2 microscope.

#### Co-immunofluorescence and fluorescent protein imaging of the chromatin tracing section

Frozen sections were first balanced at room temperature for 10 min, followed by hydration in Dulbecco’s phosphate-buffered saline (DPBS) for 5 min twice. Tissue sections were permeabilized with 0.5% (vol/vol) Triton X-100 in DPBS for 30 min at room temperature and washed twice in DPBS for 2 min. Tissue sections were then blocked for 30 min at room temperature in blocking buffer containing 1% (wt/vol) BSA (Sigma-Aldrich, A9647-100G), 22.52 mg ml^−1^ glycine (AmericanBio, AB00730), 10% (vol/vol) donkey serum (MilliporeSigma, S30–100 ml), 5% (vol/vol) goat serum (Invitrogen, 31873) and 0.1% (vol/vol) Tween-20 in DPBS. Tissue sections were incubated with rabbit anti-SPC antibody (MilliporeSigma, AB3786, 1:50) and rat anti-CD45 antibody (BioLegend, 103101, 1:100) in blocking buffer at 4 °C overnight. The samples were washed thrice in DPBS for 5 min and then incubated with DyLight 800-labeled donkey anti-rabbit secondary antibody (Invitrogen, SA5-10044, 1:1,000) and Alexa Fluor 647-labeled goat anti-rat secondary antibody (Invitrogen, A-21247, 1:1,000) in blocking buffer for 1 h at room temperature. The samples were washed thrice in DPBS for 5 min and then incubated with DAPI (Thermo Fisher Scientific, 62248) at 1:1,000 dilution in 2× saline-sodium citrate buffer (SSC) for 10 min. The samples were then mounted onto a Bioptechs FCS2 flow chamber and replenished with freshly prepared imaging buffer with an oxygen scavenging system (50 mM Tris–HCl pH 8.0, 10% wt/vol glucose, 2 mM Trolox (Sigma-Aldrich, 238813), 0.5 mg ml^−1^ glucose oxidase (Sigma-Aldrich, G2133), 40 μg ml^−1^ catalase (Sigma-Aldrich, C30)). The imaging buffer was covered with a layer of mineral oil (Sigma-Aldrich, 330779) in the reservoir tube to prevent continuous oxidation. We then selected multiple fields of view (FOVs) at predefined tumor regions based on tumor grades of the adjacent whole-section fluorescence images. Tumor grades were confirmed independently by two investigators (S.S.A. and M.D.M.). At each FOV, we sequentially took z-stack images of DAPI, GFP fluorescence, tdTomato fluorescence, anti-SPC immunofluorescence and anti-CD45 immunofluorescence with 405-nm, 488-nm, 560-nm, 647-nm and 750-nm laser illuminations. The z-stacks (7–10 μm total) had a 200-nm step size and a 0.4 s exposure time per step.

#### Primary probe hybridization

After imaging, tissue sections were de-assembled from the chamber, treated with 1 μg ml^−1^ proteinase K (Invitrogen, AM2546) in 2% (vol/vol) sodium dodecyl sulfate in 2× SSC at 37 °C for 10 min, and washed twice in DPBS for 2 min. Tissue sections were then treated with 0.1 M HCl for 5 min and washed twice in DPBS for 2 min. Tissue sections were digested with 0.1 mg ml^−1^ RNase A (Thermo Fisher Scientific, EN0531) in DPBS for 45 min at 37 °C and washed twice with 2× SSC for 2 min. Tissue sections were treated with prehybridization buffer containing 50% (vol/vol) formamide (Sigma-Aldrich, F7503) and 0.1% (vol/vol) Tween-20 in 2× SSC. Synthesized primary probes were dissolved in 25 μl hybridization buffer containing 50% (vol/vol) formamide and 20% (vol/vol) dextran sulfate (MilliporeSigma, S4030) in 2× SSC. The total probe concentration was 30–40 μM. Coverslips were immersed in hybridization buffer containing probes in 60 mm petri dishes, heat denatured in a 90 °C water bath for 3.5 min and subsequently incubated at 47 °C in a humid chamber for 36–48 h. Samples were washed with 50% (vol/vol) formamide in 2× SSC for 15 min twice followed by 2× SSC for 15 min all at room temperature. Washed samples were then incubated with 0.1-μm yellow–green beads (Invitrogen, F8803) resuspended in 2× SSC as fiducial markers for drift correction, washed with 2× SSC briefly, incubated with DAPI at 1:1,000 dilution in 2× SSC for 10 min for image registration and washed again with 2× SSC for 2 min twice.

#### Readout probe hybridization and imaging

After the primary probe hybridization, the sample was repeatedly hybridized with adaptors and readout probes, imaged and photobleached for 50 rounds. Each adaptor probe was 60 nucleotides (nt) in length, containing a 20-nt primary probe binding region and two consecutive 20-nt readout probe binding regions. Readout probes were 30-nt oligos conjugated with Alexa Fluor 647 (or Cy5) or ATTO 565 (or Cy3) fluorophores with 20-nt complementary to the readout probe binding regions of adaptors (Supplementary Table 3). To perform automatic buffer exchange, we used a Bioptechs FCS2 flow chamber and a computer-controlled, custom-built fluidics system^11,72^. First, we used DAPI images for registration to images taken before primary probe hybridization. We selected the same FOVs as those selected during co-immunofluorescence and fluorescent protein imaging. At each FOV, we took z-stack images with 488-nm and 405-nm laser illuminations for fiducial beads and DAPI, respectively. For each round of readout probe hybridization, we flowed 2 ml of readout probe hybridization buffer (20% vol/vol ethylene carbonate (Sigma-Aldrich, E26258) in 2× SSC) containing two adaptors each at 50 nM through the chamber and incubated for 15 min at room temperature. We flowed through 2 ml wash buffer (20% vol/vol ethylene carbonate in 2× SSC) for 2 min and 2 ml readout probe hybridization buffer containing two dye-labeled readout probes each at 75 nM, with 15 min incubation at room temperature. We further flowed through 2 ml wash buffer for 2 min and 2 ml imaging buffer. At each FOV, dye-labeled readout probes were imaged in 647-nm and 560-nm channels and fiducial beads were imaged in the 488-nm channel. The z-stacks (7–10 μm total) had a 200-nm step size and a 0.4 s exposure time per step. After imaging, we switched buffer to readout probe hybridization buffer containing 1 μM dye-free readout probes (blocking oligos) and photobleached with continuous simultaneous laser illuminations with 750-nm, 647-nm and 560-nm lasers for 40 s. We then flowed 5 ml 2× SSC to wash away unbound blocking oligos before the next hybridization round. A total of 50 hybridization rounds was performed.

### Arrayed RNAi screen of cell proliferation

Lentiviral infection conditions were optimized in 96-well plates for initial cell seeding number, lentiviral dosage, antibiotic concentration and assay time. KP and *K-MADM-Trp53* LUAD cells were seeded in complete cell culture media at a density of 250 cells per well in a 96-well plate, incubated for 24 h, and infected with lentiviral supernatant of Sigma Mission shRNAs targeting CPDs obtained from Yale Cancer Center Functional Genomics Core. A total of 200 μl of media was added to each well, comprising 50 μl of lentiviral supernatant and 150 μl of complete cell culture medium with 10 μg ml^−1^ polybrene (EMD Millipore, TR-1003-G). Each shRNA hairpin was tested in triplicate. All lentiviral infections were performed in duplicate to rule out the influence of lentiviral dosage on cell growth—one replicate with 6 μg/ml puromycin selection and the other replicate with no puromycin selection. After 24 h of lentiviral incubation, corresponding wells were treated with or without puromycin selection for 48 h. The cells were then incubated with complete cell culture media for 4–5 days. Total viable cell count was determined with CellTiter-Glo Luminescent Cell Viability Assay (Promega, G7572) using a Promega luminescence plate reader. For data analysis, we deducted luminescence readout values of blank wells from those of test wells. We then normalized luminescence readout values of each target shRNA hairpin to those of nontargeting control shRNA sequence (shNTC). All shRNA hairpin sequences are provided in Supplementary Table 6.

### Rnf2 targeted degradation

Two million cells of the KP LUAD cell line 31671 were nucleofected (Amaxa) with 2 μg homology-directed repair (HDR) template and 2 μM RNP (IDT Alt-R CRISPR–Cas9 sgRNA) for Rnf2-dTAG. The HDR template contained a 555-nt 5′ homology arm homologous to the 5′ end of the Rnf2 stop codon, a 30-nt linker and two HA tags, an FKBP^F36V^ and mScarlet insert (Supplementary Table 6; amplified from a gift plasmid from D. Schatz at Yale University) and a 999-nt 3′ homology arm homologous to the 3′ end of the Rnf2 stop codon. After transfection, the cells were seeded into 6-well plates and cultured for 24–48 h before FACS sorting. mScarlet+ cells were sorted into 96-well plates. Single colonies were manually picked, genotyped with PCR and validated with sequencing. The spacer sequence for Rnf2 sgRNA is GACTTTATTATGCACCCACCA. The dTAG-13 ligand and negative control ligand were added to the cells at a final concentration of 500 nM. The cells were incubated at 37 °C with 5% CO_2_.

### Image analysis

All image analyses were performed with MATLAB R2019b.

The genome-wide chromatin tracing image analysis pipeline consists of the following steps: color correction, drift correction, nucleus segmentation, foci fitting, decoding and trace linking. Detailed methods are listed in the Supplementary Note.

### Data analysis and statistics

#### Decompaction

To compare levels of chromatin compaction, we defined a decompaction score as the mean interloci distance between a locus pair on a chromosome of a cell state.

#### Heterogeneity

To compare chromatin conformation heterogeneity, we defined a heterogeneity score as the coefficient of variation (COV) of interloci distances between a locus pair on a chromosome of a cell state (variation among different chromosome copies). Similarly, the heterogeneity scores for cell pairs were defined as the root mean square of interloci distance difference normalized by the mean.

#### Demixing

To compare levels of chromatin intermixing, we defined a demixing score as the s.d. of all normalized mean interloci distances (mean interloci distance normalized to the mean of all mean interloci distances on the chromosome) on a chromosome of a cell state. The normalization accounted for and excluded the influence of chromatin compaction on demixing measurements.

#### A and B compartment polarization analysis

To identify A and B compartments, we adapted a previous algorithm to determine A and B compartment scores^11,24^. In brief, we generated a mean interloci distance matrix for each chromosome, normalized the mean distance to the expected distance calculated by power-law fitting of spatial versus genomic distances and calculated the Pearson correlation coefficient among each column pair of the normalized matrix. Finally, we applied a principal component analysis (PCA) of the Pearson correlation matrix and used coefficients of the first principal component as compartment scores. We calculated correlations between compartment scores and an averaged profile of histone H3 lysine 4 monomethylation (H3K4me1), H3K4me3, DNase I hypersensitivity site and gene densities^78,79^, and flipped signs of compartment scores if necessary, so that A and B compartment genomic regions had positive and negative scores, respectively. We then used a previously described polarization index metric to quantify the polarization of A and B compartments^24^. For chromosome 6 in Fig. 3d, we used the first 30 target genomic regions to calculate the polarization index to match target region numbers in different chromosomes. We downloaded H3K4me1 and H3K4me3 chromatin immunoprecipitation sequencing peaks and DNA DNase I hypersensitivity sites from The Encyclopedia of DNA Elements (ENCODE)—ENCFF536DWZ, ENCFF508WEP and ENCFF268DLZ. The gene density profile was downloaded from the UCSC table browser.

#### Radial score analysis

To calculate the radial score of each genomic region in each single cell, we measured the mean spatial distance among each region to the centroid of all target regions and normalized the distance to the average spatial distances from all genomic regions to the centroid.

#### The ‘Trace2State’ pipeline for single-cell chromatin conformation-based cell state visualization and classification

First, we constructed an input matrix where each row represented a single cell and each column represented the scA/B score of each genomic region^35^. The scA/B score was calculated as the mean A–B compartment score of all its spatially adjacent genomic regions within a 1,200-nm 3D neighborhood. Only cells with at least 10 traces were analyzed. Missing values were replaced with 0s. The matrix was used as input in a PCA, and the first 50 principal components were used for downstream visualizations. We scaled the 50 principal components with the PCA().fit-transform function in Python 3.11.12, and visualized the single-cell data with *t*-distributed stochastic neighbor embedding, uniform manifold approximation and projection and pairwise controlled manifold approximation^37–39^. To classify different cell states with supervised machine learning, we supplied the Classification Learner App of MATLAB with the same input scA/B score matrix and applied fivefold cross-validation to prevent overfitting. All machine learning models were trained, and the model with the highest prediction accuracy (medium Gaussian support vector machine, regularization included with box constraint level = 1) was retained to plot the confusion matrix and receiver operating characteristic curves.

#### The ‘Trace2Biomarker’ pipeline for CPD gene identification

To identify CPD genes, we first identified marker genomic regions with substantially changed scA/B scores during LUAD progression. Specifically, we extracted cells with more than 325 genomic regions. We then performed rank normalization (using the tiedrank function in MATLAB) of scA/B scores in each cell. We performed Wilcoxon rank-sum tests of rank-normalized scA/B scores of each genomic region comparing AdenomaG and LUAD. Genomic regions with *P* < 0.1 were defined as marker genomic regions. We then defined CPD genes as genes located in marker genomic regions with increased scA/B scores and higher expression (mean expression count > 10, fold change > 3, false discovery rate (FDR) < 0.05) in LUAD versus AdenomaG cells. We defined control genes as those with increased expression (mean expression count > 10, fold change > 3, FDR < 0.05) in regions with unchanged scA/B scores (*P* ≥ 0.1). Genes with increased expression only are defined as those with increased expression (mean expression count > 10, fold change > 3, FDR < 0.05) in LUAD versus AdenomaG cells, irrespective of scA/B score changes. Equal gene numbers (top 21 upregulated by fold change) were used for each gene list above and interrogated in TCGA patient survival analyses.

#### The ‘Trace2Regulator’ pipeline for 3D genome regulator identification

To identify chromatin regulators that bind to genes in marker loci, we first identified genes with increased expression (FDR < 0.1, fold change > 1) in genomic regions with increased scA/B scores (*P* < 0.1) from AdenomaG to LUAD cells. We then input the gene list into BART^55,56^ to generate chromatin regulators predicted to bind to the input genes, using default parameters. We further knocked down candidate chromatin regulators with shRNAs in the KP LUAD cell line and performed DNA MERFISH. We further compared 3D genome alterations upon the candidate chromatin regulator knockdown with those during the AdenomaG-to-LUAD progression. Candidate regulators showing concordant 3D genome alterations were identified as 3D genome regulators.

### CUT&RUN experiment and analysis

#### CUT&RUN protocol

CUT&RUN was performed using the Epicypher CUTANA ChIC/CUT&RUN Kit (14-1048) according to the manufacturer’s specifications with the following conditions and modifications: for binding to the Concanavalin beads, 500,000 cells per sample were prepared, counted twice using a hemacytometer, and averaged. Nuclei were prepared according to the Epicypher Cut and Run Manual Appendix. Nuclei were incubated with activated beads. For *Escherichia coli* spike-in DNA, 0.1 ng was added to each sample. The following antibodies (0.5 μg of antibody per reaction) were used: IgG control (CUTANA Kit Rabbit IgG CUT&RUN Negative Control Antibody), Rnf2 antibody (Cell Signaling Technology, 5694), H3K4me3 antibody (EpiCypher, 13-0041), H3K27me3 antibody (Cell Signaling Technology, 9733), H2AK119ub antibody (Cell Signaling Technology, 8240), BMI1 antibody (Active Motif, 39993) and RNA Pol II p-ser5 (Abcam, ab193467).

#### Library preparation

Libraries were prepared using Epicypher CUTANA CUT&RUN Library Prep Kit (14-1001) according to the manufacturer’s specifications with the following modification: SPRI-select beads were used after library preparation to perform 200–700 bp size selection to enrich for DNA fragments from CUT&RUN and remove adaptor dimers or high molecular weight DNA. Library quality was analyzed using Agilent Tapestation D1000 High Sensitivity Tapes (5067–5584). Sequencing was performed with 10 million reads per sample, 150 bp paired-end reads, on an Illumina NovaSeq 6000.

#### CUT&RUN data analysis

CUT&RUN reads were analyzed for quality control using FastQC (https://www.bioinformatics.babraham.ac.uk/projects/fastqc/) and trimmed using Trimmomatic. Reads were then aligned to mm9 and K-12 *E. coli* genome U00096.3 by Bowtie2 2.3.4. Picard 2.27.4 was used to downsample the reads so that the read depths of *E. coli* sequences from different samples matched each other. SAMTools 1.11 was then used to convert to BAM format, index, isolate uniquely mapped paired reads and remove duplicates. MACS2 2.2.7.1 was used to call sample narrow peaks, using IgG as an input. Read counts across genomic intervals and peak visualization were performed using deepTools 3.3 (ref. ^80^), and .bw files were visualized using Integrative Genomics Viewer.

Other detailed methods are included in the Supplementary Note.

### Statistics and reproducibility

Statistical tests and sample sizes are reported in the corresponding sections. The number of replicates was determined to be the maximum number that could be collected with the available equipment and personnel during the study period. Our study analyzed AT2 cells, AT2-derived cancer cells and immune cells expressing the pan-immune marker CD45. Other cells are excluded from the analyses. Random subsampling was performed to show that as few as 100 cells per cell state were sufficient to recapitulate the 3D genome organization changes for most structural features in lung. The investigators were not blinded to allocation during experiments and outcome assessment. We visually inspected data distributions for each statistical test, but normality and equal variances were not formally tested. Wilcoxon tests were used to avoid normality assumptions.

### Reporting summary

Further information on research design is available in the Nature Portfolio Reporting Summary linked to this article.

## Online content

Any methods, additional references, Nature Portfolio reporting summaries, source data, extended data, supplementary information, acknowledgements, peer review information; details of author contributions and competing interests; and statements of data and code availability are available at 10.1038/s41588-025-02297-w.

## Supplementary information

Supplementary Information Supplementary Note and Supplementary Fig. 1.

Reporting Summary

Supplementary Tables 1–7 Supplementary Table 1: Template oligonucleotide probe libraries for genome-wide (Supplementary Table 1-1) and fine-scale (Supplementary Table 1-2) chromatin tracing and RNA MERFISH (Supplementary Table 1-3). Supplementary Table 2: Codebooks for all target genomic loci in genome-wide chromatin tracing (Supplementary Table 2-1) and RNA MERFISH (Supplementary Table 2-2), and coordinates of genomic features in genome-wide chromatin tracing (Supplementary Tables 2-1,2-3) and in fine-scale chromatin tracing (Supplementary Table 2-4). In ‘Supplementary Table 2-1’, the first column indicates which chromosome each target genomic region in genome-wide chromatin tracing is located in. The second and third columns indicate the start coordinate and end coordinate of the target genomic region. Each region corresponds to a topologically associating domain (TAD). The fourth column indicates the binary barcode for each genomic region. ‘Supplementary Table 2-2’ contains the gene name and binary barcode of each RNA MERFISH target gene. ‘Supplementary Table 2-3’ contains mitogen-activated protein kinase (MAPK) pathway gene, oncogene and tumor suppressor gene names and coordinates of super-enhancers (mm9) in all target TADs in genome-wide chromatin tracing. Each row corresponds to a target TAD in Supplementary Table 2-1. ‘Supplementary Table 2-4’ contains gene names and genomic coordinates of each target region (mm10) in fine-scale chromatin tracing. Supplementary Table 3: Oligo sequences for adaptors, common readout probes, blocking oligos and primers. The spreadsheet contains the following eight blocks: ‘large-scale adapter’, ‘fine-scale adapter’, ‘large- and fine-scale readout’, ‘blocking oligo’, ‘large- and fine-scale primer’, ‘RNA MERFISH adapter’, ‘RNA MERFISH readout’ and ‘RNA MERFISH primer’. ‘Large-scale adapter’ contains the sequences of all 100 adaptor oligos that can bind to the primary probes in genome-wide chromatin tracing and correspond to the 100 bits in the codebook. ‘Fine-scale adapter’ contains the sequences of loci-specific adaptor probes and gene-specific adaptor probes. ‘RNA MERFISH adapter’ contains sequences of the 16 adaptor oligos that can bind to the RNA MERFISH primary probes and correspond to the 16 bits in the codebook. ‘Large- and fine-scale readout’ and ‘RNA MERFISH readout’ contain the sequences of dye-conjugated readout probes that bind the corresponding adaptors. ‘Blocking oligo’ contains dye-free oligos that have the same sequences as the common readout oligos to block any unbound sites of the previous adaptors in genome-wide chromatin tracing, fine-scale chromatin tracing and RNA MERFISH. ‘Large- and fine-scale primer’ and ‘RNA MERFISH primer’ contain the forward and reverse primer sequences for the template probe library amplification and primary probe synthesis. Supplementary Table 4: Whole-exome sequencing analysis of lung tumors from *K-MADM-Trp53* mice. Mutation calls of variants (variant allele fraction > 5%) for adenoma and LUAD tumors (*n* = 6 tumors per group). This table includes mouse number (sample), variant chromosome, genomic position, reference base (REF), variant base (ALT), variant allele fraction (Tumor_AF), number of reference read (AD[0]) and variant reads (AD[1]) in tumor and normal, gene, and protein-altering effect (missense, splice variant, stop_gained). Supplementary Table 5: RNA-seq analysis of lung tumors from *K-MADM-Trp53* mice. Differential expression analysis (DESeq2) of large green tumors derived from *K-MADM-Trp53* mice and classified as AdenomaG and LUAD based on marker gene expression. For each gene, log_2_ normalized expression counts (DESeq2) for each sample, average expression counts across all samples, log_2_ fold change (LUAD versus AdenomaG), *P* value and FDR (padj) are shown. Supplementary Table 6: shRNA sequences for lentiviral transduced stable cell lines to knock down candidate progression driver genes and sequence of the FKBPF36V and mScarlet insert. The table contains two blocks. Block 1 contains sequences for all the shRNAs of the candidate genes, positive controls and negative controls. The first column contains the gene names. The second column contains the shRNA sequences. The third column contains the hairpin numbers. Block 2 contains sequences for the FKBPF36V and mScarlet insert used for Rnf2 targeted degradation. Supplementary Table 7: List of candidate progression driver genes. References (PubMed IDs (PMID)) are listed for genes with prior literature evidence for a functional role in lung cancer pathogenesis.

## Source data

Source Data Fig. 1 Statistical source data.

Source Data Fig. 2 Statistical source data.

Source Data Fig. 3 Statistical source data.

Source Data Fig. 4 Statistical source data.

Source Data Fig. 5 Statistical source data.

Source Data Fig. 6 Unprocessed western blots.

Source Data Fig. 6 Statistical source data.

Source Data Extended Data Fig. 1 Statistical source data.

Source Data Extended Data Fig. 2 Statistical source data.

Source Data Extended Data Fig. 3 Statistical source data.

Source Data Extended Data Fig. 4 Statistical source data.

Source Data Extended Data Fig. 5 Statistical source data.

Source Data Extended Data Fig. 6 Statistical source data.

Source Data Extended Data Fig. 7 Statistical source data.

Source Data Extended Data Fig. 8 Statistical source data.

Source Data Extended Data Fig. 9 Statistical source data.

Source Data Extended Data Fig. 10 Unprocessed western blots.

Source Data Extended Data Fig. 10 Statistical source data.

## Data Availability

The snRNA-seq and bulk RNA-seq data are available from Gene Expression Omnibus (GEO; GSE275588, GSE295452). The CUT&RUN data are available from GEO (GSE295857). The whole-exome sequencing data are available from SRA (PRJNA1255157). The Hi-C and analyzed chromatin tracing data are deposited to the 4D Nucleome portal (https://data.4dnucleome.org/publications/e83173f7-95e4-40ad-9748-e13b2a518fc6/#expsets-table). Analyzed imaging and sequencing data are available at https://campuspress.yale.edu/wanglab/Cancer3DGenome/. Source data are provided with this paper.

## Code availability

Open-source code for imaging data collection is available at https://github.com/ZhuangLab/storm-control. All original codes generated for this study are available at https://campuspress.yale.edu/wanglab/Cancer3DGenome/ and on Zenodo via 10.5281/zenodo.15527545 (ref. ^81^).
