# Supplementary material for: Tracing the evolution of single-cell 3D genomes in Kras-driven cancers
Source: Nat Genet. 2025 Aug 18;57(12):3075–87. doi: 10.1038/s41588-025-02297-w (PMC12695640; doi:10.1038/s41588-025-02297-w)
Supplement: Supplementary file 1 — Supplementary Note and Supplementary Fig. 1. [file 41588_2025_2297_MOESM1_ESM.pdf]

nature genetics

Supplementary information https://doi.org/10.1038/s41588-025-02297-w

Tracing the evolution of single-cell 3D genomes in Kras-driven cancers

In the format provided by the authors and unedited

Supplementary Note

Advantages and limitations of chromatin tracing

Chromatin tracing offers several unique advantages among single-cell 3D genomics methods[1–7] in studying the cancer 3D genome. First, as an imaging-based approach, it retains the native tissue architecture *in situ*, which is essential for pathologic analyses in cancer studies and allows analyses of cell-cell interactions in shaping the 3D genome within the tumor microenvironment (**Extended Data Fig. 9**). Second, chromatin tracing directly traces the 3D folding path of chromatin, whereas sequencing-based approaches indirectly infer 3D chromatin folding based on contact events. Third, the chromatin tracing method allows integration with multiplexed fluorescent protein/immunofluorescence imaging in the same single cells, enabling cell type-specific identification (*e.g.*, SPC+ AT2 cells, CK19+ duct cells) and lineage labels (*e.g.*, MADM), whereas the potential for similar multiplexing is limited in sequencing-based methods. Finally, the chromatin tracing approach achieves higher cell throughput due to its cost efficiency. For example, a recent single-cell split-pool recognition of interactions by tag extension (scSPRITE) study[5] profiled 1,000 cells at 40-kb to 1-Mb resolution, while our work analyzed over 61,000 cells at 5-kb to 5-Mb resolution. Meanwhile, chromatin tracing has its own limitations. Since it relies on targeted profiling of genomic regions, the genomic coverage is usually low. Unlike sequencing-based approaches, chromatin tracing is unable to identify single nucleotide variants and copy number variations *de novo*. Furthermore, the genomic resolution is limited to ~2-kb and finer resolution is currently prohibited by probe length and density. Finally, it remains technically challenging to perform parallel sample imaging and data collection. On balance, chromatin tracing offers a distinctive window into cancer 3D genomic evolution *in situ* with single-cell resolution.

Probe design and synthesis in genome-wide chromatin tracing

Template probe design: In genome-wide chromatin tracing, to select target genomic regions for mouse Chr1-Chr19, we first downloaded topologically associating domain (TAD) coordinates in mouse embryonic stem cells from http://3dgenome.fsm.northwestern.edu/download.html. Second, we selected target genomic regions containing one or more of the following features: (1) Mitogen-activated protein kinase (MAPK) pathway genes; (2) "classic" oncogenes and tumor suppressor genes; and (3) super-enhancers. Third, to select other target genomic regions, we selected 30 regions equally spaced along each entire chromosome and removed those within an interval to the feature-containing target genomic regions. We then combined the remaining other target regions and feature-containing target regions as the target genomic regions. We designed about 400 oligos per 60-kb target genomic region. A total of 50 target genomic regions were designed for Chr6 and 18-25 target genomic regions were designed for each of the other autosomes. A total of 473 target genomic regions spanning Chr1-Chr19 were selected. To distinguish the identity of each target genomic region, we adapted a previously published Hamming weight 2 (HW2) binary barcode design strategy[8]. We rearranged the binary code assignment to (1) minimize the variance of the number of genomic regions imaged per round across different imaging rounds for each chromosome to make sure no more than one genomic region was imaged per imaging round for each chromosome, except for Chr6; and (2) for Chr6, maximize the genomic distance between the genomic regions imaged in the same imaging round. The template oligos for the 473 target genomic regions were designed as follows: each template oligo consisted of 6 regions (from 5' to 3'): (1) a 20-nucleotide (20-nt) forward priming region; (2) a 20-nt adapter binding region; (3) a 40-nt genome targeting region; (4) a second 20-nt adapter binding region; (5) a third 20-nt adapter binding region; and (6) a 20-nt reverse priming region. The forward and reverse 20-nt priming

sequences were generated from random sequences that were screened to lack homology to the mouse genome and optimized for polymerase chain reaction (PCR) amplifications. The 20-nt adapter binding sequences bind to 60-nt adapter oligos consisting of one 20-nt adapter sequence and two 20-nt readout probe binding sequences. Both the adapter and readout probe binding sequences were generated from random sequences with minimum homology to the mouse genome and maximum performance in signal-to-noise ratios. The 40-nt genome-targeting sequences were designed with OligoArray2.1[9] using the following parameters: (1) the melting temperatures of target sequences were between 65 °C and 85 °C; (2) the melting temperatures of potential secondary structures were less than 76 °C; (3) the melting temperatures of potential cross-hybridizations were less than 72 °C; 4) the GC content of the sequences was between 20% and 90%; (5) no consecutive repeats of six or more A's, T's, C's and G's were identified; and (6) adjacent oligos were allowed to have 30-nt overlapping sequences. Genome targeting sequences were then screened against the mouse genome with BLAST+ version 2.9.0 to ensure single matches to the reference genome[10]. Genomic targeting sequences were further screened against a mouse repetitive database from Repbase (https://www.girinst.org/repbase/)[11]. Sequences with more than 16-nt homology to a list of repetitive sequences in the mouse repetitive database were removed. A monolayer of 400 oligos were then selected for each target genomic region to construct the template probe library. The template probe library pool was purchased from Twist Biosciences. Template probe sequences are provided in **Supplementary Table 1**. Genome coordinates (mm9), codebook and target genomic region features are provided in **Supplementary Table 2**.
Primary probe synthesis: Primary probes were synthesized from the template probe library using previously published protocols following a procedure of limited-cycle PCR, *in vitro* transcription, reverse transcription, and probe purification[12–15]. All PCR primers, reverse transcription primers, adapters, and readout probes were purchased from Integrated DNA Technologies (IDT). The sequences are provided in **Supplementary Table 3**.

Probe design in fine-scale chromatin tracing

In fine-scale chromatin tracing, we designed probes that target the regulatory regions of 30 genes/genomic regions. Although we designed probes targeting 30 genes/genomic regions, with a total of 1,178 target loci (**Supplementary Tables 1-2**), we focused our analyses on the 3D chromatin organization of 15 genes encompassing 13 CPDs and known driver genes *Kras* and *Myc* (**Extended Data Fig. 8**). Genomic regions of interest were chosen based on gene annotations and previously published Hi-C data in mES cells[16]. Putative enhancers were identified by the union of ENCODE predicted enhancers and H3K4me1 (ENCFF536DWZ) and DNaseI (ENCFF268DLZ) intersected ChIP-seq peaks in adult mouse lung. We selected 40 consecutive 5-kb to 20-kb genomic loci (20 loci for *Foxa3* due to shorter genomic length) for each gene. We performed 50 rounds of three-color imaging to decode the identity of each locus. For the first 40 rounds, each 5-kb to 20-kb genomic locus was read out one at a time on all genes in each hybridization round. The last 10 rounds read out one gene at a time in the three channels. We designed 150 oligos per 5kb genomic region. The template oligos were designed as follows: each template oligo consisted of 6 regions (from 5' to 3'): (1) a 20-nt forward priming region; (2) a 20-nt genomic loci readout region; (3) a 30-nt genome targeting region; (4) a second 20-nt genomic loci readout region; (5) a 20-nt gene readout region; and (6) a 20-nt reverse priming region. The forward and reverse 20-nt priming sequences and readout sequences were generated as described above. The 30-nt genome targeting sequences were designed with ProbeDealer[17] using the following parameters: (1) the melting temperatures of the target sequences were between 66 °C and 100 °C; (2) the melting temperatures

of potential secondary structures were less than 76 °C; (3) the melting temperatures of potential cross-hybridizations were less than 72 °C; 4) the GC contents of the sequences were between 30% and 90%; (5) no consecutive repeats of six or more A's, T's, C's and G's were identified; and (6) adjacent oligos were allowed to have 20-nt overlapping sequences. Genome targeting sequences were then screened against the mouse genome with BLAST+ version 2.9.0 to ensure single matches to the reference genome[10]. Genomic targeting sequences were further screened against a mouse repetitive database from Repbase (https://www.girinst.org/repbase/)[11]. Sequences with more than 16-nt homology to a list of repetitive sequences in the mouse repetitive database were removed. A monolayer of 150 oligos per 5-kb genomic region was then selected for each target locus to construct the template probe library. The template probe library pool was purchased from Agilent. Template probe sequences are provided in **Supplementary Table 1**. Genome coordinates (mm10) are provided in **Supplementary Table 2**. Primary probes were synthesized following the protocols described above.

Coverslip treatment

Prior to tissue sectioning, coverslips (Bioptechs, 40-mm-diameter, #1.5) were first silanized as previously described[18,19]. In brief, coverslips were immersed into a 1:1 mixture of 37% (vol/vol) hydrochloric acid (HCl) and methanol at room temperature for 30 min. Coverslips were then washed with deionized water three times, followed by a 70% ethanol wash. Coverslips were dried in a 70 °C oven for 1 h and immersed into chloroform containing 0.2% (vol/vol) allyltrichlorosilane (Sigma, 107778) and 0.1% (vol/vol) triethylamine (Millipore, TX1200) for 30 min at room temperature. Coverslips were then washed with chloroform and ethanol and dried in a 70 °C oven for 1 h. Silanized coverslips can be stored in a desiccated chamber at room temperature for weeks. For tissue attachment, silanized coverslips were treated with 1% (vol/vol) polyethylenimine (Sigma, 408727) in water for 5 min and washed twice in water. Coverslips were then air-dried and ready for tissue attachment.

Mouse *K-MADM-Trp53* lung tissue chromosome 19 paint

The chromosome 19 paint experiment in mouse *K-MADM-Trp53* lung tissues was performed in combination with SPC and CD45 immunofluorescence imaging and GFP/tdTomato fluorescence imaging, following a similar procedure as described in "*Mouse K-MADM-Trp53 lung tissue chromatin tracing experiments*" except for the following. First, the primary probe library targeting mouse chromosome 19 was the same as one published previously[20]. Second, primary probes were synthesized with Alexa Fluor 647-labeled reverse transcription primers, so all probes were labeled with Alexa Fluor 647 fluorophores on the 5' end. Samples were hybridized with primary probes in petri dishes at 37 °C in a humid chamber for 36 - 48 hours. Samples were washed with 0.1% (vol/vol) Tween-20 in 2× SSC for 15 min twice at 60 °C, and once at room temperature. Samples were then washed with 2× SSC, incubated with DAPI and directly imaged without secondary probe hybridization.

Mouse *K-MADM-Trp53* lung tissue fine-scale chromatin tracing

In fine-scale chromatin tracing, we first hybridized primary probes to the genome. The hybridization procedure for fine-scale chromatin tracing is similar to that of large-scale chromatin tracing except that (1) heat denaturation was performed in a 90 °C water bath for 4 min; and (2) samples were hybridized with primary probes in petri dishes at 40 °C in a humid chamber for 36-48 hours. We then sequentially hybridized dye-labeled readout probes and performed 50 rounds of

three-color imaging, including 40 rounds for locus-specific readout and 10 rounds for gene-specific readout. The sequences of adapter and readout probes are provided in **Supplementary Table 3**.

Mouse *K-MADM-Trp53* pancreas tissue chromatin tracing experiments

The experimental procedure for the pancreas tissue is similar to that of the lung tissue except that pancreas tissue sections were permeabilized with 0.5% (vol/vol) Triton X-100 (Sigma-Aldrich, T8787) in DPBS for 20 min at room temperature, washed in DPBS for 2 min twice, incubated with rabbit anti-Cytokeratin 19 antibody (Abcam, ab52625, 1:50) in blocking buffer at 4 °C overnight. Heat denaturation was performed in a 90 °C water bath for 3 min.

Mouse cell line chromatin tracing experiments

***Stable Rnf2 knockdown and rescue cell line construction**:* Lentiviral supernatant for Sigma Mission^{TM} shRNAs targeting *Rnf2* and a non-targeting control (shNTC) was obtained from the Yale Cancer Center Functional Genomics Core. KP cells were seeded in 6-well plates (Falcon, 353046) with a density of 50,000 cells/well. Cells were infected with lentivirus at different titers 24 hours after seeding. Stable transfected cells were selected with 6 μg/mL puromycin (Gibco, A11138-03) for 48-72 hours after lentiviral transfection. Cell pellets were collected after 48 hours of selection for quantitative reverse transcription polymerase chain reaction (RT-PCR) validation and after 72 hours of selection for western blot validation. For Rnf2 rescue experiments, Rnf2 WT and Rnf2 I53S cDNAs (both harboring a mutated Rnf2 shRNA target seed sequence) were synthesized from Genscript and inserted into the multiple cloning site of pLV-EF1a-IRES-Hygro (Addgene 85134). To construct rescue cell lines (shNTC+empty vector, shRnf2+empty vector, shRnf2+I53S Rnf2), stable Rnf2 knockdown KP cells were seeded in 6-well plates (Falcon, 353046) with a density of 50,000 cells/well. Cells were infected with lentivirus at different titers 24 hours after seeding. Stable transfected cells were selected with 700 μg/mL hygromycin (Gibco, 10687010) for 48-72 hours after lentiviral transduction. Rnf2 protein expression was confirmed by western blot.

***Primary probe hybridization**:* Cells were washed with DPBS twice for 2 min each, fixed with 4% (vol/vol) paraformaldehyde (EMS, 15710) in DPBS for 10 min, and washed twice with DPBS for 2 min each. Cells were permeabilized with 0.5% (vol/vol) Triton-X in DPBS for 10 min and washed twice with DPBS for 2 min each. Next, the cells were treated with 0.1 M HCl for 5 min at room temperature, washed with DPBS twice for 2 min each, treated with 0.1 mg/mL RNase A in DPBS for 45 min at 37 °C, and washed twice with 2× SSC for 2 min each. The cells were subsequently incubated in pre-hybridization buffer containing 50% (vol/vol) formamide and 0.1% (vol/vol) Tween-20 in 2× SSC. Synthesized primary probes were dissolved in 25 μL hybridization buffer containing 50% (vol/vol) formamide and 20% (vol/vol) dextran sulfate in 2× SSC. The final probe concentration was 30-40 μM. We then added the hybridization buffer containing probes to a 60 mm petri dish and flipped the coverslip onto it so that the cells were immersed into hybridization buffer. Heat denaturation was performed by incubating the petri dish in a 90 °C water bath for 4 min. The petri dish was incubated at 47 °C in a humid chamber for 36-48 hours. After hybridization, the cells were washed with 50% (vol/vol) formamide in 2× SSC for 15 min twice at room temperature and washed with 2× SSC for an additional 15 min. We then incubated each sample with 0.22-μm light yellow beads (Spherotech, FP-0245-2) resuspended in 2× SSC as fiducial markers for drift correction and washed the sample with 2× SSC briefly.

***Readout probe hybridization and imaging**:* We followed the same "Readout probe hybridization and imaging" procedure, as performed in "*Mouse K-MADM-Trp53 lung tissue chromatin tracing experiments*" described in **Methods**, except that (1) DAPI images were acquired after sequential readout probe hybridization and imaging; (2) both readout probe hybridization buffer and wash buffer were composed of 35% (vol/vol) formamide in 2× SSC instead of 20% (vol/vol) ethylene carbonate; (3) fiducial beads were imaged in the 405-nm channel; and (4) tris(2-carboxyethyl)phosphine (TCEP; Sigma-Aldrich, C4706) cleavage was used instead of photobleaching for fluorescence signal removal between the readout hybridization rounds. TCEP reduces disulfide bonds connecting fluorophores to readout probes, removing fluorophores from readout probes. The TCEP cleavage buffer was composed of 50 mM TCEP and 1 μM dye-free readout probes in 20% (vol/vol) ethylene carbonate to block unoccupied readout probe binding regions on adapters from interfering with the next round of hybridization and imaging.

Mouse cell line RNA MERFISH experiment

***Probe design**:* The RNA MERFISH probe design followed a previously published procedure[20]. The template oligos were designed as follows: each template oligo consisted of six regions (from 5' to 3'): (1) a 20-nt forward priming region; (2) a 20-nt readout region; (3) a 30-nt RNA transcript targeting region; (4) a second 20-nt readout region; (5) a third 20-nt readout region; and (6) a 20-nt reverse priming region. The sequences of the forward and reverse priming regions and readout regions were generated as described above. The 30-nt RNA transcript targeting sequences were designed with ProbeDealer[17] using the following parameters: (1) the melting temperatures of the target sequences were between 71 °C and 100 °C; (2) the melting temperatures of potential secondary structures were less than 76 °C; (3) the melting temperatures of potential cross-hybridizations were less than 72 °C; 4) the GC contents of the sequences were between 30% and 90%; (5) no "GGGG/CCCC/TTTTTT/AAAAAA" was identified; (6) targeting sequences were not allowed to overlap with each other. Targeting sequences were then screened against the mouse repetitive database and spliced transcriptome database (GENCODE, M35) with BLAST+ version 2.9.0 to ensure each sequence is non-repetitive and is transcribed from only one gene[10]. The mouse repetitive database was downloaded from Repbase (https://www.girinst.org/repbase/)[11]. The template probe library pool was purchased from Twist Biosciences. Template probe sequences are provided in **Supplementary Table 1**.

Primary probe hybridization**:* The LUAD cell line isolated from the *K-MADM-Trp53* mouse model (SA6082inf) cultured on a coverslip was washed with DPBS twice for 2 min each, fixed with 4% (vol/vol) paraformaldehyde in DPBS for 10 min, and washed twice with DPBS for 2 min each. Cells were permeabilized with 0.5% (vol/vol) Triton-X in DPBS for 10 min and washed twice with DPBS for 2 min each. Next, the cells were incubated in pre-hybridization buffer containing 50% (vol/vol) formamide and 0.1% (vol/vol) Tween-20 in 2× SSC with 2000× diluted murine RNase inhibitor (New England Biolabs, M0314L) for 5 min. Synthesized primary probes (by procedures in the ***Primary probe synthesis section) were dissolved in 25 μL hybridization buffer comprising 50% formamide, 0.1% wt/v yeast tRNA (Life Technologies, 15401011), 10% dextran sulfate (Sigma, D8906-50G) and 100× diluted murine RNase inhibitor in 2× SSC. The final probe concentration was 30-40 μM. We then added the hybridization buffer containing probes to a 60 mm petri dish and flipped the coverslip onto it so that the cells were immersed into hybridization buffer. The petri dish was incubated at 37 °C in a humid chamber for 24-28 hours. After hybridization, the cells were washed with 0.1% (vol/vol) Tween 20 in 2xSSC at 60 °C for 15 min each, and an additional 15 min at room temperature. We then applied 0.1-μm yellow-green

beads (Invitrogen, F8803) resuspended in 2× SSC as fiducial markers for sample drift correction during sequential hybridization as described for chromatin tracing.

Microscope setup

For imaging, we used a custom-built microscope with a Nikon Ti2-U body, a Nikon CFI Plan Apo Lambda 60× Oil (NA1.40) objective lens, and an active auto-focusing system[21]. Different laser settings were applied for imaging tissues or cell lines. For *K-MADM-Trp53* lung and pancreas chromatin tracing experiments, illumination lasers included: a 750-nm laser (2RU-VFL-P-500-750-B1R, MPB Communications), a 647-nm laser (2RU-VFL-P-1000-647-B1R, MPB Communications), a 560-nm laser (2RU-VFL-P-1000-560-B1R, MPB Communications), a 488-nm laser (2RU-VFL-P-500-488-B1R, MPB Communications), and a 405-nm laser (OBIS 405 nm LX 50 mW, Coherent). The five laser lines were directed to the sample using a multi-band dichroic mirror (ZT405/488/561/647/752rpc-UF2, Chroma) on the excitation path. Laser intensities were controlled with an acousto-optic tunable filter (AOTF, 97-03309-01 Gooch & Housego), and laser on-off was controlled by mechanical shutters (LS3S2Z0, Vincent Associates). For cell line chromatin tracing experiments, we used a Lumencor CELESTA light engine for illumination, with the following laser wavelengths: 405-nm, 477-nm, 546-nm, 638-nm, and 749-nm. The lasers were directed to the sample using a corresponding penta-band dichroic mirror from Lumencor. Laser intensities and on-off were controlled by internal controls of the light engine. The 750/749-nm laser was used to excite and image DyLight 800-conjugated donkey anti-rabbit secondary antibody. The 647/638-nm laser was used to excite and image Alexa Fluor 647 (or Cy5) on readout probes and on the anti-rat secondary antibody. The 560/546-nm laser was used to excite and image tdTomato fluorescence and ATTO 565 (or Cy3) on readout probes. The 488/477-nm laser was used to excite and image GFP fluorescence and the yellow-green fiducial beads for drift correction. The 405-nm laser was used to excite and image the DAPI stain and the light-yellow fiducial beads. On the emission path, we had a multi-band emission filter (ZET405/488/561/647-656/752-nm Chroma for the tissue imaging setup or a corresponding penta-band emission filter for the cell line imaging setup) and a Hamamatsu Orca Flash 4.0 V3 camera. The pixel size of our system was 107.9 nm. To automatically scan and image multiple FOVs, we used a computer-controlled motorized x–y sample stage (SCAN IM 112×74, Marzhauser). For z-stepping and active auto-focusing, a piezo z positioner (Mad City Labs, Nano-F100S) was used.

Bulk RNA-sequencing of lung tumors

Large green tumors from *K-MADM-Trp53* mice were microdissected under a Nikon SMZ1270 fluorescence dissection stereo microscope and flash frozen. Tissue was pulverized using a BioPulverizer that was sprayed down with RNAse Away (Molecular BioProducts) and cooled with liquid nitrogen. RNA and genomic DNA were extracted using the AllPrep DNA/RNA Mini Kit (Qiagen). Library preparation for RNA-sequencing was performed by the Yale Center for Genome Analysis (YCGA), and libraries were sequenced on a NovaSeq S2 (Illumina) to obtain 100-bp paired-end reads. All reads that passed FASTQC quality metrics were mapped to the UCSC mm10 mouse genome and normalized gene count matrices were generated through STAR v2.7.9. Further analysis after trimming, alignment, and normalization were performed using DESeq2 on R[22]. Hierarchical clustering was done through the pheatmap package. Genes upregulated ($\log_2$ fold change > 2, FDR < 0.05) or downregulated ($\log_2$ fold change < -1, FDR < 0.05) in LUAD compared to AdenomaG were compared to the MSigDB Hallmarks gene set collection (https://www.gsea-msigdb.org/gsea/msigdb) to determine enrichment (hypergeometric test).

Normalized expression counts and differential expression analyses are included in **Supplementary Table 5**.

Whole exome sequencing analysis of copy number and single nucleotide variants

Tumor genomic DNA was obtained from flash frozen tumors using the AllPrep DNA/RNA Mini Kit, as described above. Paired normal DNA was obtained by extraction from formalin-fixed paraffin-embedded (FFPE) slides of the same mouse lung by YCGA. Whole exome sequencing (WES) was performed using the Mouse All Exon kit (Agilent) for target capture followed by next-generation sequencing by Psomagen. Mouse tumor samples were sequenced at 200× read coverage while healthy lung tissue was sequenced at 50×. For copy number analysis, sample reads were mapped to the GRCm38 reference genome with BWA-MEM (v 0.7.17)[23], sorted based on coordinates with Picard SortSam tools (http://broadinstitute.github.io/picard/), and indexed with Samtools[24,25]. We estimated copy number variation by first calculating the total number of mapped reads on each chromosome in each sample, normalized by the sequencing depth. We then divided the normalized readout counts in each adenoma/LUAD to those in matched normal lung tissue from the same mouse to calculate the relative copy number of each chromosome in comparison to normal. We then doubled the values to account for the diploid nature of the normal mouse genome. For single nucleotide variant (SNV) analysis, after initial quality control and trimming the raw sequences using fastp (v0.23.2)[26], the trimmed sequence data were mapped to the mouse reference genome UCSC mm10 using BWA-MEM[23]. Duplicate reads were identified by employing the MarkDuplicates tool from the Genome Analysis Toolkit (GATK)/picard. Base Quality Score Recalibration (BQSR) was performed using BaseRecalibrator & ApplyBQSR with reference to the dbSNP database and data from the Sanger Mouse Genetics Programme (Sanger MGP). We created a panel of normals (PoN) containing germline and artifactual sites by running Mutect2 in tumor-only mode for each of 12 normal samples. We constructed a GenomicsDB datastore from the normal Mutect2 calls. The normal calls were combined to create the PoN with CreateSomaticPanelOfNormals. In this way, not only were the matched normal variants filtered out, but also any variants present in other normal mouse samples. We employed Mutect2 in GATK4 (v 4.4.0.0)[27] to call somatic variants in a tumor/normal variant calling pipeline. The variant calling also utilized the PON file created using the normal calls. To address potential orientation biases in the raw data, we applied the LearnReadOrientationModel tool to learn the orientation bias model. Then, we filtered the unprocessed variants using FilterMutectCalls. We applied additional filtering criteria to reduce the false-positive rate of the variants identified. The positions in either tumor or matched normal samples with <10× coverage were removed from further analysis. At least three reads were required to support variants called in tumor samples, with no more than zero reads for the variant allele in the matched normal. A variant allele fraction of >5% was used to make mutation calls, which are listed in **Supplementary Table 4**.

Single-nucleus RNA sequencing

***Single-nucleus isolation and RNA sequencing library preparation*:** Nuclei from dissected lung tumors (two biologic replicates) were isolated by adapting a previously reported protocol[28]. Briefly, a stock solution of 2× salt-Tris buffer (ST buffer) composed of 292 mM sodium chloride (NaCl) (Thermo Fisher, BP358), 20 mM Trizma-HCl (Sigma, T2194-100ML), 2 mM calcium chloride ($CaCl_2$) (VWR, E506-100ML), and 42 mM magnesium chloride ($MgCl_2$) (Alfa Aesar, J62411) in nuclease-free water (Invitrogen, 10977-15) was prepared fresh before isolation. 0.02% NP-40 Substitute based ST lysis buffer (NST lysis buffer) was generated using 1 mL of 2× ST buffer, 4

μL of 10% NP-40 Substitute (Sigma, 98379), 10 μL of BSA (NEB, B9000S), 20 μL of Superase-In RNase inhibitor (Invitrogen, AM2696), and 966 μL of nuclease free water. Resuspension buffer was also freshly prepared using 880 μL of 1× Dulbecco's PBS (Sigma, D8537-500ML), 100 μL of BSA, and 20 μL of Superase-In RNase inhibitor. 50 μL of NST lysis buffer was added to flash frozen tissue in a 1.5 mL microcentrifuge tube. The sample was continuously minced on ice with Noyes Spring scissors (Fine Science Tools, 15514-12) for four minutes to isolate nuclei. Additional NST lysis buffer was added to the sample for a final volume of 0.5 mL and passed through a 30 μm MACS SmartStrainer (Miltenyi Biotec, 130-098-458) into a 15 mL conical tube. The sample was then washed by adding 4 mL of 1x ST buffer through the strainer. Samples were spun down in a swinging bucket centrifuge for 5 min at 500 g at 4°C and resuspended in 50-100 μL of Resuspension buffer depending on the size of the pellet. The nuclei suspension was then passed through a 35-μm filter (Falcon, 352235). Nuclei were counted on a hemacytometer, and 10,000 nuclei were loaded onto a 10x Chromium chip for Chromium Single Cell 3' Library (V3, PN-1000075) generation.

Single-nucleus RNA sequencing data analysis: Libraries were sequenced according to 10x Chromium manufacturer recommendations. The reads were aligned to the mm10-2020-A reference transcriptome to include introns using Cell Ranger count (v.7.1.0; 10x Genomics). To remove ambient RNA, raw matrices generated from Cell Ranger were inputted into Cellbender (Snapshot 11) using remove-background and run on the Terra platform with an FPR set to 0.01. Doublets were detected using Scrublet (v0.2.1) via doublet_detection (Snapshot 2) on Terra. Seurat_5.0.5 was used for downstream analyses. Cell barcodes with (1) 500-5000 genes; (2) 1000-10,000 transcript unique molecular identifiers (UMIs); and (3) less than 10% mitochondrial counts were included in the analysis. Data were normalized with a global scaling "LogNormalize" method and a scale factor of 10,000. We performed feature selection with the "vst" method and 2,000 features, scaled the data with all genes, performed principal component analysis (PCA) for dimensionality reduction, and clustered single nucleus gene expression with the Louvain algorithm. For tumor analyses, we first excluded fibroblasts and fibrocytes (*Ptprc, Cd163, S100a4, S100a8, S100a9, Cd90, Col1a1, Il6, Ccl3, Ccl4*), endothelial cells (*Pecam1, Cdh5, Tie2, Foxf1*), and immune cells (*Mrc1, Trac, Jchain, Ighg1*) and further identified adenoma cells (*Sftpc, Lyz2, Cxcl15, Hopx*), and LUAD cells (*Eif2s3y, Chsy3, Ldlrad4, Large1*) based on their marker gene expression patterns[28–30]. Single-cell copy number of each gene was estimated with the InferCNV 1.14.2 package[31]. The copy number heterogeneity of each gene was calculated by the coefficient of variation of the estimated gene copy number across all single cells.

Western Blot

Cells were trypsinized, harvested, and lysed with radioimmunoprecipitation assay (RIPA) buffer (ThermoFisher, 89900) containing 1× protease inhibitors (ThermoFisher, 87786) at 4 °C for 30 min. Cell lysate supernatant was collected after centrifugation at 16,000 × g for 20 min at 4 °C. Supernatants were quantified using the bicinchoninic acid (BCA) protein assay kit (Pierce, 23225). A total of 20 μg protein was denatured at 95 °C for 5 min and loaded on a 4-20% precast polyacrylamide gel (BioRad, 4568094) for gel electrophoresis. Proteins were transferred onto a polyvinylidene fluoride (PVDF) membrane (ThermoFisher, IB24001) with an iBlot2 gel transfer device (Invitrogen, IB21001). The PVDF membrane was blocked with 5% (vol/vol) BSA in 1× Tris-buffered saline with Tween-20 (TBST) (AmericanBio, AB14330-01000), incubated with primary antibodies at 4 °C overnight, washed with 1× TBST for 5 min for three times, incubated with horseradish peroxidase (HRP)-conjugated secondary antibodies at room temperature for 1

hour, and washed three times with 1× TBST for 5 min. Membranes were treated with SuperSignal West Pico Plus chemiluminescent substrate (ThermoScientific, 34577) and imaged with a ChemiDoc imaging system (BioRad). For fluorescence detection, proteins were transferred onto a nitrocellulose membrane (BioRad, 1620145) with the Trans-Blot Turbo transfer system (BioRad). Blots were washed once with 1x PBS (Boston BioProducts, BM-220X), blocked for an hour with Intercept Blocking Buffer (LiCOR, 927-7001), and incubated with primary antibodies overnight at 4°C. Blots were subsequently washed three times with 1x PBS-0.1% Tween20 for 10 min (Sigma-Aldrich, P1379-500ML), incubated with fluorescence secondary antibodies at room temperature for 45 minutes, washed three more times with 1x PBS-0.1% Tween20 followed by 1x PBS once prior to ChemiDoc imaging. The following antibodies were used: rabbit anti-Rnf2 (Cell Signaling Technologies, 5694S, 1:500), mouse anti-Hsp90 (BD Biosciences, 610418, 1:10,000), HRP-conjugated goat anti-rabbit IgG (Abcam, ab6721, 1:3000), HRP-conjugated goat anti-mouse IgG (BioRad, STAR207P, 1:10,000), DyLight 800 4X PEG-conjugated goat anti-mouse IgG 800 (Cell Signaling Technologies, 5257S, 1:10,000), and DyLight 680-cojugated goat anti-rabbit IgG (Cell Signaling Technologies, 5366S, 1:10,000).

Image analysis

DAPI registration: GFP and tdTomato fluorescence images and SPC and CD45 co-immunofluorescence images were aligned to genome-wide chromatin tracing images using intensity-based image registration of DAPI images. To process DAPI images, we first took the average z-projection of each DAPI image stack and normalized it to its maximum intensity. We then reduced the background by normalizing the average projection image to the background calculated by the adaptthresh function. We then adjusted the threshold of the image so that the maximum and minimum intensities corresponded to the 3rd and 1st quartiles of the pixel intensities. We then applied an "opening-by-reconstruction" technique with a disk-shaped morphological structural element with a radius of 25 pixels to reduce the noise. Next, to align the processed DAPI images, we applied an intensity-based image registration algorithm. We used the imregtform function to estimate the geometric transformation for image alignment and the imregconfig function to generate the optimizer and metric configurations used by imregtform. We first optimized an initial transformation condition, and then used the optimized initial conditions to improve image alignment. For initial condition optimizations, we reduced the InitialRadius of the optimizer (generated by imregconfig) by a scale factor of 5 and set the MaximumIterations of the optimizer to 500. We then applied the optimizer and metric to the imregtform function with the "similarity" geometric transformation option to generate the initial geometric transformation object. We used the imregtform function to align the processed DAPI images with the "affine" geometric transformation option, the previously generated optimizer and metric, and the initial geometric transformation object. We finally generated a geometric transformation object to align DAPI images taken with fluorescent protein and co-immunofluorescence images to the DAPI images taken with the first hybridization round of genome-wide chromatin tracing.

GFP+ and tdTomato+ cell analysis: To identify cells with GFP or tdTomato fluorescence signals, we first generated maximum projections of GFP or tdTomato images along the z direction and aligned the images to the first-round readout hybridization images of genome-wide chromatin tracing. We then used an algorithm that can manually adjust the intensity threshold to determine GFP+ or tdTomato+ cells (Thresholding an image - File Exchange - MATLAB Central (mathworks.com)). We finally generated binary masks to distinguish GFP+ and tdTomato+ cells.

SPC+ and CD45+ cell extraction: To identify cells with SPC or CD45 immunofluorescence signals, we generated average projections of SPC or CD45 images along the z direction and normalized the images to the background calculated by the adaptthresh function. We then performed standard deviation filtering of the image with the stdfilt function, and filled holes with the imfill function, using a connectivity of 8 pixels. We then converted the SPC or CD45 images to binary images, used the regionprops function to identify SPC or CD45 patches, and excluded patches smaller than 150 pixels. The remaining patches were used to generated binary masks to distinguish SPC+ or CD45+ cells. Finally, to match the signals to nuclei, we generated binary masks for each cell nucleus using the DAPI images as described in the "*Nucleus segmentation*" section below and dilated the binary mask of each nucleus with a disk-shaped structural element of 10 pixels. We then multiplied each single-nucleus binary mask to the SPC or CD45 binary masks. Nuclei with more than 100 overlapping pixels were labeled as nuclei of SPC+ or CD45+ cells.

The genome-wide chromatin tracing image analysis pipeline consists of the following steps: color correction, drift correction, nucleus segmentation, foci fitting, decoding, and trace linking.
Color correction*:* The color shift between 647-nm and 560-nm laser channels was corrected by taking z-stack calibration images of Tetraspeck microspheres (0.1 µm, Invitrogen, T7279) attached to a coverslip surface. A polynomial spatial transformation structure in x and y was constructed with the cp2tform function and used for xy color shift correction. The color shift in z was corrected by calculating the mean z shift.
Drift correction*:* To correct for sample drifts between different rounds of hybridizations, we determined 3D positions (x, y, z) of fiducial beads with 3D Gaussian fitting for each hybridization round. We subtracted 3D positions (x, y, z) of the first hybridization round from each hybridization round to generate the drift correction profiles for all hybridization rounds.
Nucleus segmentation*:* Because the tissue sections largely consisted of a monolayer of cells, we segmented single cell nuclei in 2D based on DAPI staining patterns. We first applied drift corrections to the DAPI images and took their average projections along the z direction. We then normalized the DAPI average projection images to the background calculated by the adaptthresh function with a neighborhood size of 101 pixels. We further removed small "bright" objects using "opening-by-reconstruction" and small "dark" objects using "closing-by-reconstruction" techniques, both with a disk-shaped structuring element of 15 pixels. These processed DAPI images were further analyzed to extract foreground and background markers for the watershed algorithm. To obtain foreground markers for each single nucleus, we calculated the regional maxima with the imregionalmax function. To acquire background markers, we binarized the processed DAPI images and calculated their complement. We then used the imimposemin function to modify the processed DAPI images so that the regional minima occurred at foreground and background marker pixels. Finally, we applied the watershed function to the modified DAPI images for nucleus segmentation. We excluded small debris (<300 pixels) and under-segmented doublet nuclei (>9000 pixels) from our analyses.
Foci fitting*:* To determine the intensity threshold for DNA foci identification, we adapted a previously developed adaptive thresholding procedure so that the fitted foci number matched the expected DNA loci count[20]. We then fitted 3D positions (x, y, z) of all DNA foci using a 3D radial center algorithm[32] in each bit. We further applied color correction and drift correction to the fitted DNA foci in each bit, so that all fitted DNA foci were in the same 3D coordinate system as the first bit in the 560-nm laser channel. The signal intensity of each fitted DNA spot was normalized to the median signal intensities of all DNA foci in the corresponding image.

Decoding and trace linking: After we generated all fitted DNA foci in each bit in single nuclei, we adapted a previously reported expectation-maximization procedure for decoding[8]. First, we identified all valid spot pairs corresponding to a valid barcode whose two fitted DNA spots were within 500-nm spatial distance. For each spot pair, we calculated three quality metrics: (1) the distance between the 3D positions (x, y, z) of the spot pairs; (2) the difference between the signal intensities of the spot pairs; and (3) the average signal intensity of the spot pairs. We then calculated the percentages of spot pairs with worse qualities than a given spot pair (e.g. larger distance, larger intensity difference, smaller intensities) and calculated the product of the three percentages as the quality score of the given spot pair. Next, for all spot pairs containing the same repetitive spot, we retained one spot pair with the highest quality score. Then, for each target genomic locus, we retained the top four spot pairs with the highest quality scores in each nucleus. After we obtained all processed spot pairs in all nuclei in each FOV, we linked the DNA loci positions into traces using a previously developed symmetric nearest neighbor approach[20]. We identified the 3D centroid positions of each linked chromosome territory and calculated a fourth quality metric: the distance between each spot pair to the nearest corresponding chromosome territory centroid. We then iteratively updated the quality scores, removed repetitive spot pairs, retained the top four spot pairs per locus with the highest quality scores, and performed trace linking. For each single nucleus, if more than 99% (iteration rounds no more than 10) or 97% (iteration rounds more than 10) of spot pairs in the current iteration were the same as the previous iteration, the nucleus would be labeled as “decoded”. If more than 85% of nuclei in the FOV were labeled as “decoded”, the iteration would be terminated and spot pairs in the current iteration were stored as the finalized spot pairs. To link the finalized spot pairs (detected genomic regions) into chromatin traces, we first defined initial traces using the detected genomic regions in the first hybridization round. To grow the chromatin trace, we link the detected genomic regions to the traces if the detected genomic regions in the current hybridization round are the nearest neighbor to those in the previous hybridization round, and vice versa. After linking chromatin traces, we refit the missing genomic regions of each chromatin trace using the finalized spot pairs if they are within 6 pixels to the periphery of the chromosome territory. There are a small proportion (around 10%) of chromatin traces with overlapping genomic regions after the refitting, so we further identified traces with >50% overlapping genomic regions and excluded the shorter ones. For each chromosome in a single cell, we then retained the longest two chromatin traces. The quality of each dataset was further confirmed by analyzing the detection efficiency of each target genomic region along detected traces and trace length distribution, which were consistent across all datasets and all cancer states.
Fine-scale data analysis: For fine-scale chromatin tracing data analysis, the analytical procedure is the same as that in large-scale chromatin tracing mentioned above except for decoding and trace linking. We first fitted foci as described in “*Foci fitting*” above in 50 hybridization rounds in three color channels. The expected foci count per hybridization round was set as twice the number of target genomic loci in the specific round in the color channel. To link traces, we first identified the centroid position of the gene foci in the gene hybridization round, which approximated the centroid of the chromatin trace. Next, in each of the 40 genomic locus hybridization rounds, if a genomic locus was within 500 nm of the centroid, we included it into the chromatin trace. Chromatin traces shorter than 7 loci were removed. In each cell, we retained the longest two chromatin traces for each gene. Although we collected fine-scale chromatin folding data on 30 genes/genomic regions, with a total of 1,178 target loci (**Supplementary Tables 1-2**), we focused our analyses on the 3D chromatin organization of 15 genes encompassing CPDs and known driver genes (**Extended Data Fig. 8**). To identify enhancer-promoter loops (E-P loops), we first calculated the mean spatial

distance between each pair of loci. We excluded loci in hybridization rounds with poor signal-to-noise ratios. We then calculated the expected distances, as published previously[12,20] using all cells of each state (power law fitting of mean spatial distance versus genomic distance). For each trace, we calculated the normalized distance (spatial distance/expected distance) between each pair of loci. We performed normalization for two reasons. First, given the polymer nature of chromatin traces, genomic regions with larger genomic distances in between tend to have longer spatial distances. Therefore, loop calling often requires normalization of the spatial distance against the expected distance at each genomic distance to address the confounding polymer nature of chromatin traces, as has been routinely performed in Hi-C analysis[33]. Second, there is an overall increase in compaction during progression among all the gene regions profiled in the fine-scale chromatin tracing. The normalization removes this overall compaction background to reveal the E-P looping strength on top of the background. We identified normalized distances between all loci to the promoter locus and performed one-sided Wilcoxon rank-sum tests and false discovery rate (FDR) multiple comparison correction to identify loci with lower normalized distances to the promoter than those of neighboring loci. If such locus contained a putative enhancer, we called it an E-P loop. We used E-P interactions in LUAD cells to identify CPD gene E-P interactions. Putative enhancers were identified by the union of Ensembl predicted enhancers and H3K4me1 (ENCFF536DWZ) and DNaseI (ENCFF268DLZ) intersected ChIP-seq peaks in adult mouse lung.

<u>Data analysis and statistics</u>

***Identification of AT2 cells spatially close to and far from immune cells**:* To distinguish AT2/cancer cells close to and far from immune cells, we extracted binary masks of nuclei of SPC+ cells (or GFP+CD45− cancer cells in LUAD tumors) and of CD45+ cells and dilated the SPC+/cancer cell binary mask by 50 pixels. The SPC+/cancer cells with overlapping pixels with the CD45+ mask were identified as AT2/cancer cells spatially close to immune cells, whereas SPC+/cancer cells with no overlapping pixels with the CD45+ mask were identified as AT2/cancer cells distant from immune cells.

***Probability for randomly selected genes to inhibit cell growth**:* To quantify the percentage of randomly selected genes that can affect cell growth, we downloaded the gene_dependency.csv table from https://figshare.com/articles/dataset/DEMETER_2_Combined_RNAi/9170975, which contained the probabilities that knocking down one gene had a cell growth inhibition or death effect. Cancer cell line genetic dependencies were estimated using the DEMETER2 model applied to a combination of three large-scale Cancer Dependency Map RNAi screening datasets (the Broad Institute Project Achilles, Novartis Project DRIVE, and the Marcotte et al. breast cell line dataset). We identified all mouse LUAD cell lines from the table with available RNAi data and calculated the mean probability of all genes in mouse LUAD cells. 6.4% of randomly selected genes are expected to affect cell growth.

***TCGA patient survival analysis**:* Clinical data for TCGA LUAD patient survival and RNA-seq data were obtained from the GDAC website from the Broad Institute (https://gdac.broadinstitute.org/). The identified CPD genes of LUAD were converted to human orthologs. The RNA-seq expression matrix and the gene list were applied as inputs to score the individual expression files using GSVA R package with ssGSEA scoring method[34,35]. TCGA LUAD patients were grouped based on high/low ssGSEA scores (most correlated/least correlated) using the top/bottom quintiles. Kaplan-Meier survival analysis was carried out using survfit R function with log-rank significance test.

Quantification of single-nucleus and single-cell gene expression homogeneity*:* For expression homogeneity calculation, we used a similar algorithm as described previously[30]. We first randomly subsampled 100 cells and calculated the cosine similarity of gene expression between each cell pair. We repeated the process 100 times, each time using the mean cosine similarity as the gene expression homogeneity score.

Hi-C experiment and analysis

Hi-C experiments were performed in two *K-MADM-Trp53* LUAD tumors from different mice. Hi-C data was generated using the Arima High Coverage Hi-C kit for Animal Tissues and the Arima Library Prep Module according to the Arima Genomics manufacturer's protocols. DNA was sheared to an average fragment size of 500 bp using the Covaris S220 system. Hi-C reads were aligned to the mm10 reference genome and processed with the Juicer pipeline (version 1.6) using default parameters[36]. The A and B compartments were identified with a similar pipeline as described in "*A and B compartment polarization analysis*".

Supplementary Figure

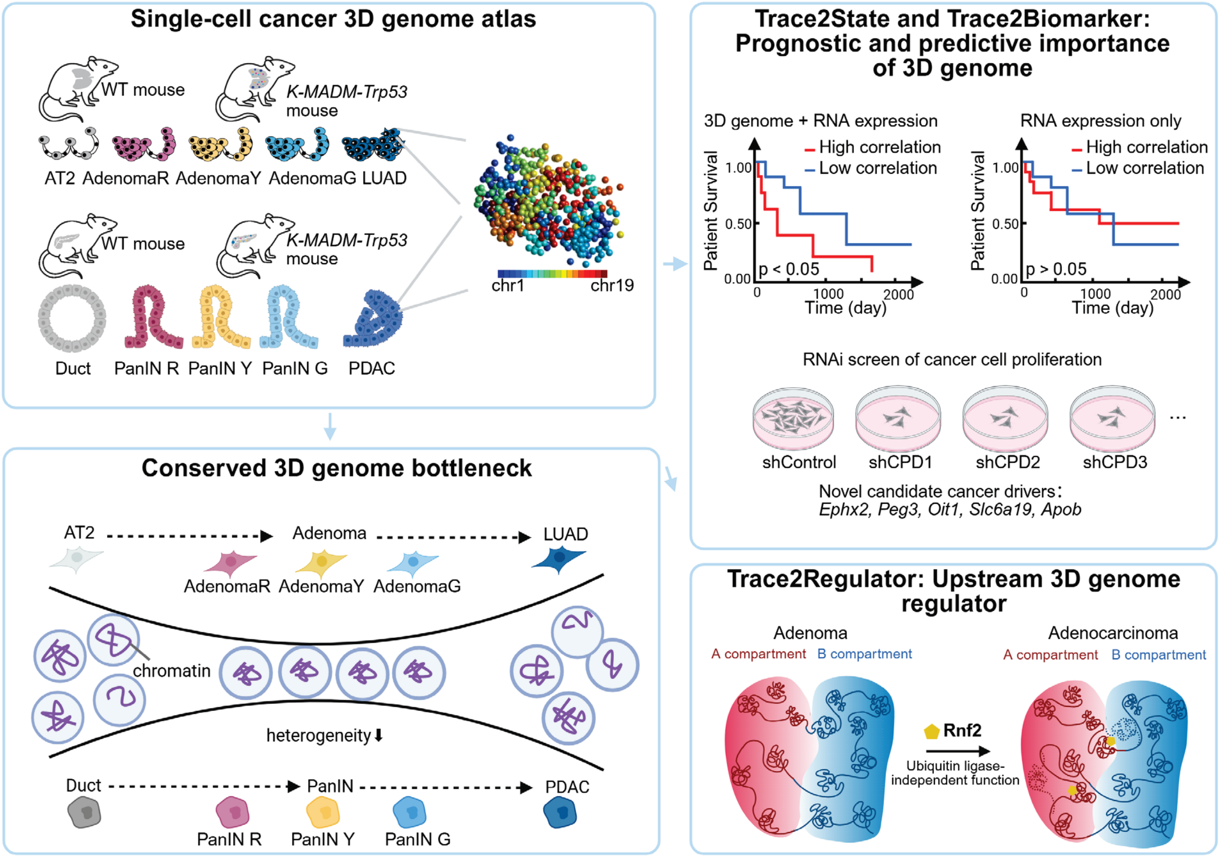

Supplementary Fig. 1. Schematic illustration of the experimental approach and major findings. In this work, we generated single-cell 3D genome atlases during lung and pancreatic cancer progression. Our data revealed stereotypical, stage-specific and conserved alterations in 3D genome folding as cancers progress from normal to preinvasive to invasive tumors, elucidating a potential structural bottleneck during early cancer progression. We developed "Trace2State" and "Trace2Biomarker" pipelines and revealed the utility of 3D genome mapping in discovering prognostic and predictive biomarkers. We further developed a "Trace2Regulator" pipeline and identified a ubiquitin ligase-independent role for Rnf2 in 3D genome regulation. The schematic was created with BioRender.

Supplementary References

1. Nagano, T. *et al.* Single-cell Hi-C reveals cell-to-cell variability in chromosome structure. *Nature* **502**, 59–64 (2013).
2. Tan, L., Xing, D., Chang, C.-H., Li, H. & Xie, X. S. Three-dimensional genome structures of single diploid human cells. *Science* **361**, 924–928 (2018).
3. Stevens, T. J. *et al.* 3D structures of individual mammalian genomes studied by single-cell Hi-C. *Nature* **544**, 59–64 (2017).
4. Flyamer, I. M. *et al.* Single-nucleus Hi-C reveals unique chromatin reorganization at oocyte-to-zygote transition. *Nature* **544**, 110–114 (2017).
5. Arrastia, M. V. *et al.* Single-cell measurement of higher-order 3D genome organization with scSPRITE. *Nat Biotechnol* **40**, 64–73 (2022).
6. Ramani, V. *et al.* Massively multiplex single-cell Hi-C. *Nat Methods* **14**, 263–266 (2017).
7. Nagano, T. *et al.* Cell-cycle dynamics of chromosomal organization at single-cell resolution. *Nature* **547**, 61–67 (2017).
8. Su, J.-H., Zheng, P., Kinrot, S. S., Bintu, B. & Zhuang, X. Genome-Scale Imaging of the 3D Organization and Transcriptional Activity of Chromatin. *Cell* **182**, 1641-1659.e26 (2020).
9. Rouillard, J.-M., Zuker, M. & Gulari, E. OligoArray 2.0: design of oligonucleotide probes for DNA microarrays using a thermodynamic approach. *Nucleic Acids Res* **31**, 3057–3062 (2003).
10. Camacho, C. *et al.* BLAST+: architecture and applications. *BMC Bioinformatics* **10**, 421 (2009).
11. Bao, W., Kojima, K. K. & Kohany, O. Repbase Update, a database of repetitive elements in eukaryotic genomes. *Mobile DNA* **6**, 11 (2015).

12. Wang, S. *et al.* Spatial organization of chromatin domains and compartments in single chromosomes. *Science* **353**, 598–602 (2016).

13. Moffitt, J. R. *et al.* High-throughput single-cell gene-expression profiling with multiplexed error-robust fluorescence in situ hybridization. *PNAS* **113**, 11046–11051 (2016).

14. Liu, M. *et al.* Chromatin tracing and multiplexed imaging of nucleome architectures (MINA) and RNAs in single mammalian cells and tissue. *Nat Protoc* **16**, 2667–2697 (2021).

15. Chen, K. H., Boettiger, A. N., Moffitt, J. R., Wang, S. & Zhuang, X. Spatially resolved, highly multiplexed RNA profiling in single cells. *Science* **348**, aaa6090 (2015).

16. Bonev, B. *et al.* Multiscale 3D Genome Rewiring during Mouse Neural Development. *Cell* **171**, 557-572.e24 (2017).

17. Hu, M. *et al.* ProbeDealer is a convenient tool for designing probes for highly multiplexed fluorescence in situ hybridization. *Scientific Reports* **10**, 22031 (2020).

18. Moffitt, J. R. *et al.* High-performance multiplexed fluorescence in situ hybridization in culture and tissue with matrix imprinting and clearing. *PNAS* **113**, 14456–14461 (2016).

19. Lu, Y. *et al.* Spatial transcriptome profiling by MERFISH reveals fetal liver hematopoietic stem cell niche architecture. *Cell Discov* **7**, 47 (2021).

20. Liu, M. *et al.* Multiplexed imaging of nucleome architectures in single cells of mammalian tissue. *Nature Communications* **11**, 2907 (2020).

21. Wang, S., Moffitt, J. R., Dempsey, G. T., Xie, X. S. & Zhuang, X. Characterization and development of photoactivatable fluorescent proteins for single-molecule–based superresolution imaging. *Proc. Natl. Acad. Sci. U.S.A.* **111**, 8452–8457 (2014).

22. Love, M. I., Huber, W. & Anders, S. Moderated estimation of fold change and dispersion for RNA-seq data with DESeq2. *Genome Biol* **15**, 550 (2014).

23. Li, H. & Durbin, R. Fast and accurate short read alignment with Burrows-Wheeler transform. *Bioinformatics* **25**, 1754–1760 (2009).

24. Li, H. *et al.* The Sequence Alignment/Map format and SAMtools. *Bioinformatics* **25**, 2078–2079 (2009).

25. Li, H. A statistical framework for SNP calling, mutation discovery, association mapping and population genetical parameter estimation from sequencing data. *Bioinformatics* **27**, 2987–2993 (2011).

26. Chen, S., Zhou, Y., Chen, Y. & Gu, J. fastp: an ultra-fast all-in-one FASTQ preprocessor. *Bioinformatics* **34**, i884–i890 (2018).

27. McKenna, A. *et al.* The Genome Analysis Toolkit: A MapReduce framework for analyzing next-generation DNA sequencing data. *Genome Res.* **20**, 1297–1303 (2010).

28. Slyper, M. *et al.* A single-cell and single-nucleus RNA-Seq toolbox for fresh and frozen human tumors. *Nat Med* **26**, 792–802 (2020).

29. Choi, J. *et al.* Inflammatory Signals Induce AT2 Cell-Derived Damage-Associated Transient Progenitors that Mediate Alveolar Regeneration. *Cell Stem Cell* **27**, 366-382.e7 (2020).

30. Marjanovic, N. D. *et al.* Emergence of a High-Plasticity Cell State during Lung Cancer Evolution. *Cancer Cell* **38**, 229-246.e13 (2020).

31. Timothy Tickle, Itay Tirosh, Christophe Georgescu, Maxwell Brown, Brian Haas. infercnv. Bioconductor https://doi.org/10.18129/B9.BIOC.INFERCNV.

32. Parthasarathy, R. Rapid, accurate particle tracking by calculation of radial symmetry centers. *Nat Methods* **9**, 724–726 (2012).

33. Yu, M. *et al.* SnapHiC: a computational pipeline to identify chromatin loops from single-cell Hi-C data. *Nat Methods* **18**, 1056–1059 (2021).

34. Barbie, D. A. *et al.* Systematic RNA interference reveals that oncogenic KRAS-driven cancers require TBK1. *Nature* **462**, 108–112 (2009).

35. Hänzelmann, S., Castelo, R. & Guinney, J. GSVA: gene set variation analysis for microarray and RNA-Seq data. *BMC Bioinformatics* **14**, 7 (2013).

36. Durand, N. C. *et al.* Juicer Provides a One-Click System for Analyzing Loop-Resolution Hi-C Experiments. *Cell Systems* **3**, 95–98 (2016).
