# Supplementary material for: Tracing the evolution of single-cell 3D genomes in Kras-driven cancers
Source: Nat Genet. 2025 Aug 18;57(12):3075–87. doi: 10.1038/s41588-025-02297-w (PMC12695640; doi:10.1038/s41588-025-02297-w)
Supplement: Supplementary file 2 — Reporting Summary [file 41588_2025_2297_MOESM2_ESM.pdf]

nature portfolio

Corresponding author(s): Mandar Deepak Muzumdar, Siyuan Wang

Last updated by author(s): Jun 1, 2025

Reporting Summary

Nature Portfolio wishes to improve the reproducibility of the work that we publish. This form provides structure for consistency and transparency in reporting. For further information on Nature Portfolio policies, see our Editorial Policies and the Editorial Policy Checklist.

Statistics

For all statistical analyses, confirm that the following items are present in the figure legend, table legend, main text, or Methods section.

n/a	Confirmed	
☐	☒	The exact sample size (*n*) for each experimental group/condition, given as a discrete number and unit of measurement
☐	☒	A statement on whether measurements were taken from distinct samples or whether the same sample was measured repeatedly
☐	☒	The statistical test(s) used AND whether they are one- or two-sided<br>*Only common tests should be described solely by name; describe more complex techniques in the Methods section.*
☐	☒	A description of all covariates tested
☐	☒	A description of any assumptions or corrections, such as tests of normality and adjustment for multiple comparisons
☐	☒	A full description of the statistical parameters including central tendency (e.g. means) or other basic estimates (e.g. regression coefficient) AND variation (e.g. standard deviation) or associated estimates of uncertainty (e.g. confidence intervals)
☐	☒	For null hypothesis testing, the test statistic (e.g. *F*, *t*, *r*) with confidence intervals, effect sizes, degrees of freedom and *P* value noted<br>*Give P values as exact values whenever suitable.*
☒	☐	For Bayesian analysis, information on the choice of priors and Markov chain Monte Carlo settings
☒	☐	For hierarchical and complex designs, identification of the appropriate level for tests and full reporting of outcomes
☐	☒	Estimates of effect sizes (e.g. Cohen's *d*, Pearson's *r*), indicating how they were calculated

Our web collection on statistics for biologists contains articles on many of the points above.

Software and code

Policy information about availability of computer code

Data collection	Data were collected using open source python 2.7.16 codes from https://github.com/ZhuangLab/storm-control.
Data analysis	MATLAB codes for raw image analysis and downstream data analysis are available at https://campuspress.yale.edu/wanglab/Cancer3DGenome/. The following softwares are used in this study for analysis: OligoArray2.1, BLAST+2.9.0, STAR v2.7.9, fastp v0.23.2, BWA-MEM v0.7.17, Python 3.11.12, Bowtie2 2.3.4, Picard 2.27.4, SAMTools 1.11, MACS2 2.2.7.1, deepTools 3.3, GATK4 4.4.0.0, DESeq2 1.32.0, Cell Ranger count 7.1.0 (10x Genomics), Scrublet 0.2.1, InferCNV 1.14.2, Juicer 1.6, and MATLAB version R2019b.

For manuscripts utilizing custom algorithms or software that are central to the research but not yet described in published literature, software must be made available to editors and reviewers. We strongly encourage code deposition in a community repository (e.g. GitHub). See the Nature Portfolio guidelines for submitting code & software for further information.

Data

Policy information about availability of data

All manuscripts must include a data availability statement. This statement should provide the following information, where applicable:

- Accession codes, unique identifiers, or web links for publicly available datasets
- A description of any restrictions on data availability
- For clinical datasets or third party data, please ensure that the statement adheres to our policy

All raw imaging data are available upon request and are not deposited online due to the prohibitively large data size. The snRNA-seq and bulk RNA-seq data have

been deposited to Gene Expression Omnibus (GEO, GSE275588, GSE295452). The CUT&RUN data have been deposited to GEO (GSE295857). The whole exome sequencing data have been deposited to SRA (PRJNA1255157). The Hi-C and analyzed chromatin tracing data have been deposited to the 4D Nucleome portal (https://data.4dnucleome.org/publications/e83173f7-95e4-40ad-9748-e13b2a518fc6/#expsets-table). Analyzed imaging and sequencing data are available at https://campuspress.yale.edu/wanglab/Cancer3DGenome/.

Research involving human participants, their data, or biological material

Policy information about studies with human participants or human data. See also policy information about sex, gender (identity/presentation), and sexual orientation and race, ethnicity and racism.

Reporting on sex and gender	This information is not involved in this study.
Reporting on race, ethnicity, or other socially relevant groupings	This information is not involved in this study.
Population characteristics	This information is not involved in this study.
Recruitment	This information is not involved in this study.
Ethics oversight	This information is not involved in this study.

Note that full information on the approval of the study protocol must also be provided in the manuscript.

Field-specific reporting

Please select the one below that is the best fit for your research. If you are not sure, read the appropriate sections before making your selection.

☒ Life sciences ☐ Behavioural & social sciences ☐ Ecological, evolutionary & environmental sciences

For a reference copy of the document with all sections, see nature.com/documents/nr-reporting-summary-flat.pdf

Life sciences study design

All studies must disclose on these points even when the disclosure is negative.

Sample size	Sample sizes are reported in the manuscript and figure legends. The number of replicates was determined to be the maximal number we could collect with available equipment and personnel during the study period. Sample sizes were not predetermined by calculation due to unknown effect sizes at the time of study design.
Data exclusions	Our study analyzed AT2 cells, AT2-derived cancer cells, and immune cells expressing the pan-immune marker CD45. Other cells are excluded in the analyses.
Replication	For lung tissue large-scale chromatin tracing experiments, we collected 16 biological replicates across multiple lung lobes (most containing multiple discrete tumors) from 13 mice at different stages of LUAD progression, harboring normal AT2, adenoma, and LUAD cells. Overall a total of 26,852 cells were analyzed. For lung tissue fine-scale chromatin tracing experiments, we collected 3 independent experimental datasets from 3 mice. A total of 12,661 cells were analyzed. For pancreas tissue large-scale chromatin tracing experiments, we collected 3 independent experimental datasets from 2 mice. A total of 2,677 cells were analyzed. For lentiviral RNAi screen, we collected 3 biological replicates for each shRNA hairpin. Each replicate has 250 cells initially seeded per well in a 96-well plate. For Rnf2 experiments, we collected at least two biological replicates for each condition. Each condition has at least 2000 cells analyzed. All attempts at replication were successful.
Randomization	Mice were not randomized, as there were no treatment groups. To confirm the robustness of our findings, we performed random subsampling to show that as few as 100 cells per cell state were sufficient to recapitulate the 3D genome organization changes for most structural features analyzed in Figs. 2 and 3.
Blinding	Not applicable. Data analysis was conducted automatically using the computational pipelines described in the paper.

Reporting for specific materials, systems and methods

We require information from authors about some types of materials, experimental systems and methods used in many studies. Here, indicate whether each material, system or method listed is relevant to your study. If you are not sure if a list item applies to your research, read the appropriate section before selecting a response.

Materials & experimental systems

n/a	Involved in the study
	☒ Antibodies
	☒ Eukaryotic cell lines
☒	Palaeontology and archaeology
	☒ Animals and other organisms
☒	Clinical data
☒	Dual use research of concern
☒	Plants

Methods

n/a	Involved in the study
☒	ChIP-seq
☒	Flow cytometry
☒	MRI-based neuroimaging

Antibodies

Antibodies used

rabbit anti-SPC antibody (Millipore, AB3786)
rat anti-CD45 antibody (BioLegend, 103101)
DyLight 800-labeled donkey anti-rabbit secondary antibody (Invitrogen, SA5-10044)
Alexa Fluor 647-labeled goat anti-rat secondary antibody (Invitrogen, A-21247)
IgG Control antibody – CUTANA Kit Rabbit IgG CUT&RUN Negative Control Antibody
RNF2 antibody – CST RING1B (D22F2) XP® Rabbit mAb #5694
H3K4me3 antibody – Epicypher Rabbit Polyclonal H3K4me3 13-0041
H3K27me3 antibody – CST Tri-Methyl-Histone H3 (Lys27) (C36B11) Rabbit mAb #9733
H3K9me3 antibody – CST Tri-Methyl-Histone H3 (Lys9) (D4W1U) Rabbit mAb #13969
H2AK119ub antibody – CST Ubiquityl-Histone H2A (Lys119) (D27C4) Rabbit mAb #8240
BMI-1 antibody – Active Motif BMI-1 antibody (mAb) 39993
RNA Pol II p-ser5 – Abcam Anti-RNA polymerase II phosphor-S5 EPR19015
Purified mouse anti-HSP90 antibody (BD, 610418)
Cytokeratin 19 antibody (Abcam, ab52625)

Validation

Antibodies were validated by the manufacturers and published studies as follows:
1. rabbit anti-SPC antibody (Millipore, AB3786). Anti-Prosurfactant Protein C (proSP-C) Antibody detects level of Prosurfactant Protein C (proSP-C) and has been published & validated for use in ELISA, Immunohistochemistry (IHC), Immunohistochemistry - Paraffin (IHC-P) in mouse lung and Western Blotting (WB).
2. rat anti-CD45 antibody (BioLegend, 103101). The antibody has been tested by immunofluorescence staining in fresh frozen mouse spleen. Cited in at least 188 publications.
3. DyLight 800-labeled donkey anti-rabbit secondary antibody (Invitrogen, SA5-10044). The antibody has been validated by western blot in mouse retina, Caco-2 cells, K-562 cells and HeLa cells.
4. Alexa Fluor 647-labeled goat anti-rat secondary antibody (Invitrogen, A-21247). The antibody has been validated by immunofluorescence staining in A549, U2OS and metastatic breast cancer blood samples.
5. IgG Control antibody – CUTANA Kit Rabbit IgG CUT&RUN Negative Control Antibody. The antibody has been validated by CUT&RUN in K562 cells.
6. RNF2 antibody – CST RING1B (D22F2) XP® Rabbit mAb #5694. This antibody has been validated using SimpleChIP® Enzymatic Chromatin IP Kits. It has been tested in immunofluorescence imaging of HeLa cells and CUT&RUN in NCCIT cells. Cited in at least 91 publications.
7. H3K4me3 antibody – Epicypher Rabbit Polyclonal H3K4me3 13-0041. This antibody meets EpiCypher's lot-specific SNAP-Certified™ criteria for specificity and efficient target enrichment in both CUT&RUN and ChIP applications. It has been tested in CUT&RUN in K562 cells and immunofluorescence imaging in HeLa cells.
8. H3K27me3 antibody – CST Tri-Methyl-Histone H3 (Lys27) (C36B11) Rabbit mAb #9733. This antibody has been validated using SimpleChIP® Enzymatic Chromatin IP Kits. It has been tested in CUT&RUN in HeLa cells and immunofluorescence imaging in HeLa cells. Cited in at least 1065 publications.
9. H3K9me3 antibody – CST Tri-Methyl-Histone H3 (Lys9) (D4W1U) Rabbit mAb #13969. This antibody has been validated using SimpleChIP® Enzymatic Chromatin IP Kits. It has been tested in flow cytometry and immunofluorescence imaging in HeLa cells. Cited in at least 112 publications.
10. H2AK119ub antibody – CST Ubiquityl-Histone H2A (Lys119) (D27C4) Rabbit mAb #8240. This antibody has been validated using SimpleChIP® Enzymatic Chromatin IP Kits. It has been tested in flow cytometry and immunofluorescence imaging in HeLa cells, and CUT&RUN in NCCIT cells. Cited in at least 298 publications.
11. BMI-1 antibody – Active Motif BMI-1 antibody (mAb) 39993. This antibody has been validated for use in ChIP and/or ChIP-Seq in mouse embryonic fibroblast cells, and can be used with Active Motif's ChIP-IT® High Sensitivity Kit or our magnetic bead-based ChIP-IT® Express Kits.
12. RNA Pol II p-ser5 – Abcam Anti-RNA polymerase II phosphor-S5 EPR19015. It has been tested in flow cytometry in HeLa cells and immunofluorescence imaging in HeLa and PC-12 cells.
13. Purified mouse anti-HSP90 antibody (BD, 610418) – Routinely tested by manufacturer and validated using lysates from HeLa cells. It has been tested in immunofluorescence imaging in WI-38 cells and western blot in HeLa cells. Cited in at least 242 publications.
14. Rabbit monoclonal [EP1580Y] to Cytokeratin 19 (Abcam, ab52625). This antibody has been validated in Flow Cyt (Intra), mIHC, ICC/IF, WB, IHC-P and reacts with mouse and human. It has been tested in immunohistochemistry in human pancreas, flow cytometry in MCF-7 and HeLa cells, and immunofluorescence in HepG2 cells, Cited in at least 195 publications.

Eukaryotic cell lines

Policy information about cell lines and Sex and Gender in Research

Cell line source(s)	The KP mouse primary lung adenocarcinoma cell line, 31671, was a gift from Dr. Nik Joshi and was derived from an autochthonous LSL-KrasG12D/KraWT,p53fl/fl mouse administered with Adeno-Cre. The K-MADM-Trp53 mouse primary lung adenocarcinoma cell line, SA6082inf, was derived from a K-MADM-Trp53 mouse. HEK-293FT, human embryonic kidney cell line, used for lentivirus production, Thermo Fisher Scientific R70007.
Authentication	The KP and K-MADM-Trp53 cell lines were derived from dissociated primary mouse lung tumors, as described above, and correct Kras and Trp53 genotype were confirmed by PCR.
Mycoplasma contamination	All cell lines tested negative for mycoplasma contamination.
Commonly misidentified lines (See ICLAC register)	No commonly misidentified lines were used in the study.

Animals and other research organisms

Policy information about studies involving animals; ARRIVE guidelines recommended for reporting animal research, and Sex and Gender in Research

Laboratory animals	Mice were housed in a specific-pathogen free facility with controlled temperature, humidity and day/night cycles and maintained in a mixed background. MADM11-GT (Stock #013749) and MADM11-TG (Stock #013751) were obtained from the Jackson Laboratory. LSL-KrasG12D and Trp53KO mice were a gift from Dr. Tyler Jacks. LSL-KrasG12D/KrasWT; MADM11-TG,Trp53KO/MADM11-TG,Trp53WT breeder mice were generated as previously described. LSL-KrasG12D/KrasWT; MADM11-TG,Trp53KO/MADM11-TG,Trp53WT were crossed with MADM11-GT mice to generate K-MADM-Trp53 experimental mice of both sexes for analysis. Ages of mice are reported below.
Wild animals	The study did not involve wild animals.
Reporting on sex	Both male and female mouse datasets were collected. No sex-specific analyses were performed. Large-scale chromatin tracing in lung included four WT mice (91-day male, 191-day female, 283-day male, 91-day female), four mice with adenoma (225-day male, 221-day male, 232-day female, 224-day male), and five mice with LUAD (232-day male, 487-day male, 494-day male, 221-day male, 551-day male). Large-scale chromatin tracing in pancreas included a WT mouse (59-day male) and a mouse with PanIN and PDAC tumors (46-day male). Fine-scale chromatin tracing in mouse lung included a WT mouse (191-day female), a mouse with adenoma (225-day male) and a mouse with LUAD (494-day male).
Field-collected samples	The study did not involve samples collected from the field.
Ethics oversight	Animal studies were approved by the Institutional Animal Care and Use Committee of Yale University.

Note that full information on the approval of the study protocol must also be provided in the manuscript.
