# Supplementary figures and images for: Tracing the evolution of single-cell 3D genomes in Kras-driven cancers
Source: Nat Genet. 2025 Aug 18;57(12):3075–87. doi: 10.1038/s41588-025-02297-w (PMC12695640; doi:10.1038/s41588-025-02297-w)

## Source Data Fig. 6a

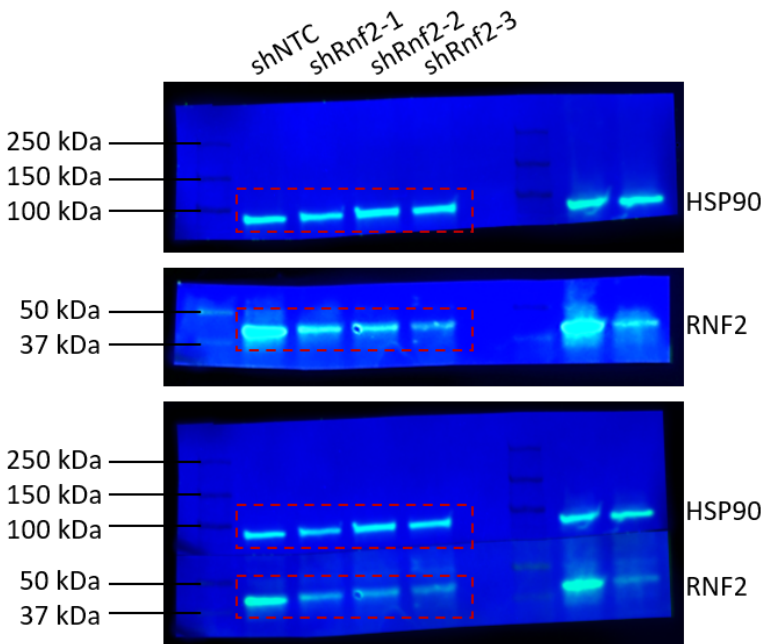

Supplement: Supplementary file 9 — Unprocessed western blots. [file 41588_2025_2297_MOESM9_ESM.pdf]

Source Data Extended Data Fig. 10a

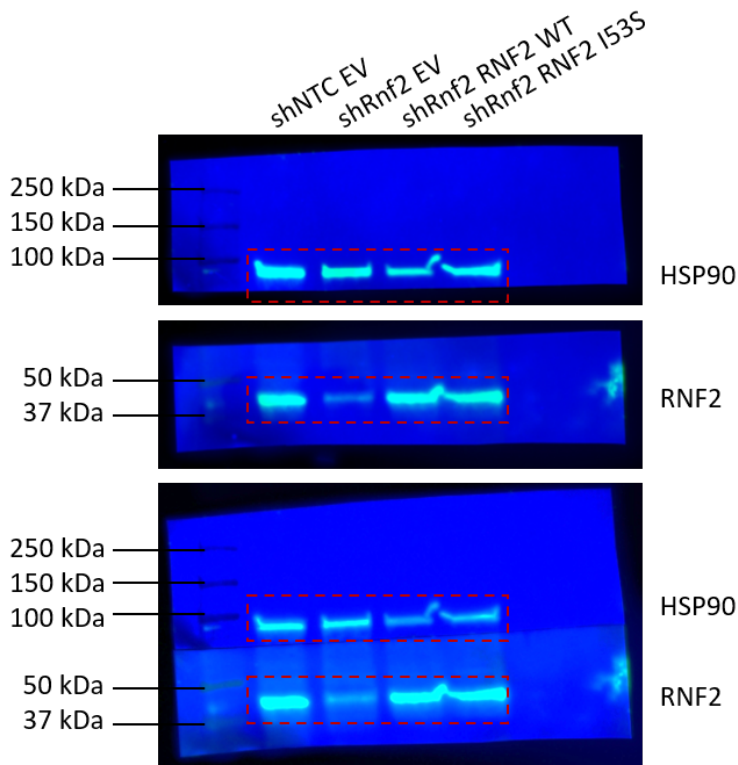

Source Data Extended Data Fig. 10c

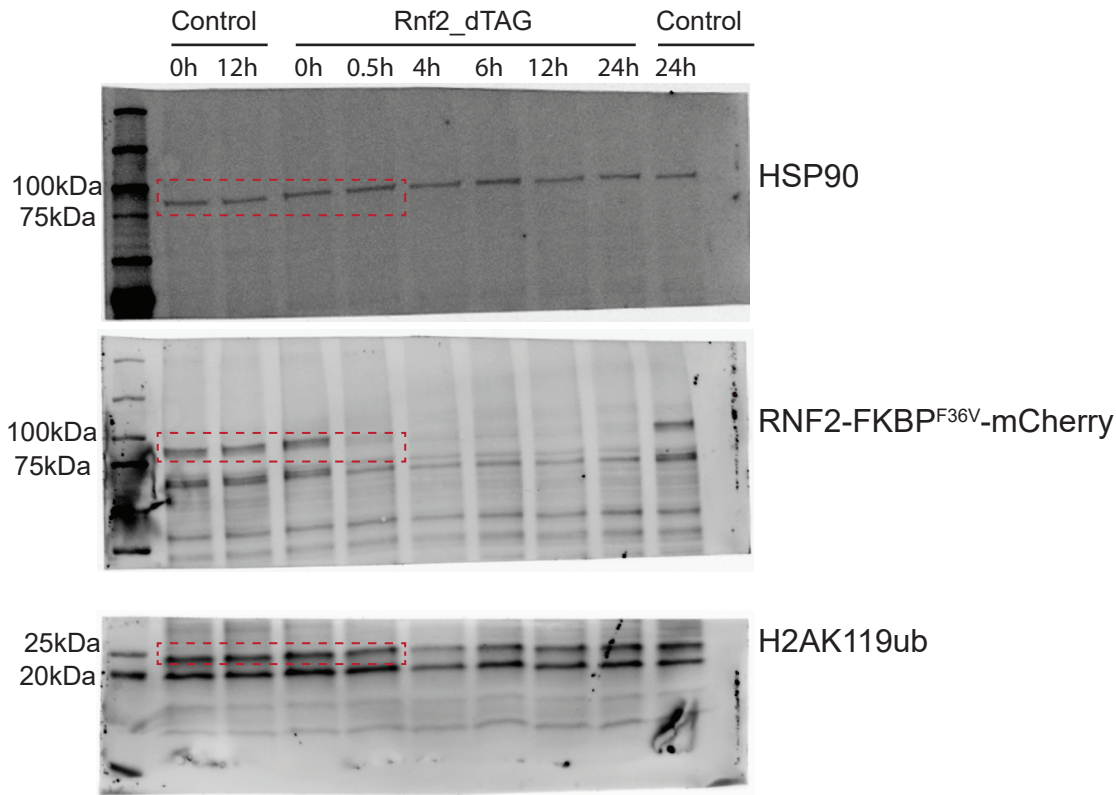

Supplement: Supplementary file 20 — Unprocessed western blots. [file 41588_2025_2297_MOESM20_ESM.pdf]
